# Spatial decoupling of CH_4_ oxidation and CO_2_ reduction enables near-stoichiometric dry reforming of methane

**DOI:** 10.1039/d6sc03014a

**Published:** 2026-06-01

**Authors:** Wenbin Li, Jiyun Ren, Wenjie Guo, Qing Guo, Sai Zhang, Yongquan Qu

**Affiliations:** a School of Chemistry and Chemical Engineering, Northwestern Polytechnical University 710072 Xi'an China zhangsai1112@nwpu.edu.cn yongquan@nwpu.edu.cn

## Abstract

The practical application of dry reforming of methane (DRM) is hindered by catalyst deactivation, primarily due to the deviation of the ideal 1 : 1 H_2_ : CO stoichiometry for competitive CH_4_ and CO_2_ adsorption/activation. Excessive CH_4_ decomposition results in H_2_ : CO > 1 with carbon deposition, while predominant CO_2_ chemisorption leads to H_2_ : CO < 1 with the favorable reverse water-gas shift (RWGS) side reaction. Herein, we demonstrate an *O-migration coupling strategy on Pt/CeO_2_ featuring Pt clusters and frustrated Lewis pairs (FLPs, consisting of two Ce^3+^ and one lattice oxygen) to achieve near-stoichiometric and durable DRM. The FLP sites on the CeO_2_ support, independent of Pt–CeO_2_ interfaces, reduce CO_2_ to CO while generating *O species. These *O species migrate to Pt clusters, driving the partial CH_4_ oxidation. Through this *O-migration-enabled spatial decoupling of CO_2_ reduction and CH_4_ oxidation, the catalyst delivers a near-stoichiometric H_2_ : CO ratio of 0.99 and an unprecedented CH_4_ conversion rate of 93.9 mol g_Pt_^−1^ h^−1^ at 700 °C. Moreover, stable performance is exhibited for over 400 h, with a turnover number exceeding 7 200 000. This work establishes oxygen migration coupling as a potential strategy for spatially decoupled redox catalysis beyond DRM.

## Abstract 2

Conceptual illustration of the spatially decoupled CH_4_ oxidation and CO_2_ reduction on the Pt clusters and FLP sites, respectively, mediated through oxygen spillover.

## Graphical abstract

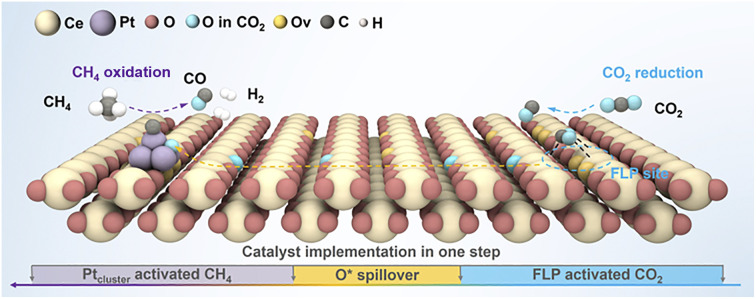

## Introduction

**Fig. 1:**
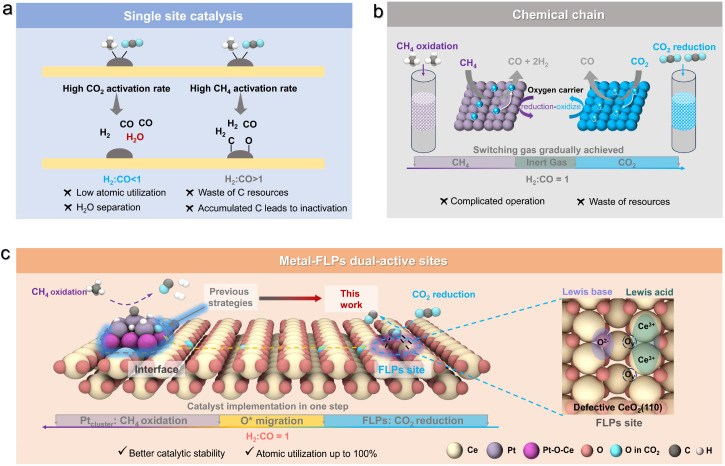
Spatially decoupled CO_2_ reduction and CH_4_ oxidation. (a) Schematic diagram highlighting the inherent limitations of conventional DRM catalysts. (b) Schematic diagram of chemical looping DRM, achieving separation of CO_2_ reduction and CH_4_ oxidation. (c) Conceptual illustration of the spatially decoupled CH_4_ oxidation and CO_2_ reduction on the Pt clusters and FLP sites, respectively, mediated through oxygen spillover.

Syngas (CO/H_2_), a pivotal platform for chemical synthesis, was projected to reach a global production capacity exceeding 290.9 million Nm^3^ per h in 2025.^1^ Dry reforming of methane (DRM) represents a particularly promising route for syngas production, offering the dual advantages of reducing carbon emissions and supplying valuable feedstock.^2–6^ However, using conventional catalysts, competitive adsorption/activation of CH_4_ and CO_2_ at the catalyst surface invariably leads to preferential conversion of one reactant, with the H_2_ : CO ratio deviating from unity.^7–11^ As illustrated in Fig. 1a, excessive CH_4_ adsorption and decomposition lead to elevated H_2_ : CO ratios (>1) and severe carbon deposition, causing catalyst deactivation.^7,12,13^ Conversely, predominant CO_2_ chemisorption triggers the reverse water-gas shift (RWGS) reaction, resulting in sub-stoichiometric H_2_ : CO ratios (<1) and compromised hydrogen utilization.^11,14–17^ Therefore, the rational design of catalysts that can balance CH_4_ and CO_2_ activation is essential to realize near-stoichiometric DRM, enabling durability and industrial-scale application.

A temporal decoupling strategy, chemical looping DRM, has been developed to address competitive adsorption by separating CH_4_ oxidation and CO_2_ reduction into alternating steps over oxygen-storage materials (Fig. 1b).^18–20^ This approach effectively suppresses coking formation and avoids RWGS, enabling near-stoichiometric H_2_ : CO ratios (∼1). Despite this promise, the practical implementation of chemical looping DRM suffers from inherent drawbacks, including energy-intensive temperature swings, reliance on inert gas purging for phase separation, and sluggish kinetics of oxygen mobility in oxygen storage materials, collectively hindering scalability and operational flexibility.

Herein, we report a rationally designed catalyst featuring dual-active sites of Pt clusters and frustrated Lewis pairs (FLPs) (Fig. 1c), where CH_4_ oxidation and CO_2_ reduction proceed concurrently yet remain spatially segregated on distinct catalytic sites, subsequently coupling through *O migration across the CeO_2_ support. The FLP sites on CeO_2_(110), composed of adjacent Ce^3+^ Lewis acids paired with a neighbouring lattice O^2−^ Lewis base (Fig. 1c),^21^ exhibit exceptional CO_2_ adsorption and activation, outperforming conventional oxygen vacancies and metal–CeO_2_ interfaces. This enables spatial decoupling of CO_2_ reduction from CH_4_ dissociation on Pt clusters. Mechanistically, CO_2_ reduction at FLP sites directly generates CO and reactive *O species; the *O then migrates rapidly across the CeO_2_ surface to Pt clusters, where it drives the partial oxidation of CH_4_ to H_2_ and CO (Fig. 1c). This dynamic coupling affords a near-stoichiometric H_2_ : CO ratio of 0.99, which in turn delivers exceptional catalytic stability (>400 h) with a turnover number exceeding 7 200 000. Moreover, the catalyst achieves a remarkable CH_4_ conversion rate of 93.9 mol g_Pt_^−1^ h^−1^ at 700 °C, surpassing recently reported state-of-the-art systems.

## Results and discussion

### Theoretical investigation and catalytic performance

Ceria (CeO_2_) serves as an ideal oxygen storage component owing to its exceptional redox properties and defect-engineered surface chemistry, which facilitates facile oxygen mobility within its lattice.^22–24^ However, the nonpolar C–H bonds of CH_4_ resist activation on the Lewis acid/base pairs of CeO_2_, necessitating metal sites where d-orbitals facilitate the requisite electron transfer for C–H scission.^17,25–28^ In contrast, CO_2_ adsorption is efficiently promoted at the metal–CeO_2_ interface.^29–31^ To achieve spatially decoupled catalysis, we reasoned that the CeO_2_ supports must possess sites with exceptionally strong and selective affinity for CO_2_, thereby isolating its activation from CH_4_ oxidation on metal sites and preventing *H intermediates from triggering the deleterious RWGS reaction.

**Fig. 2:**
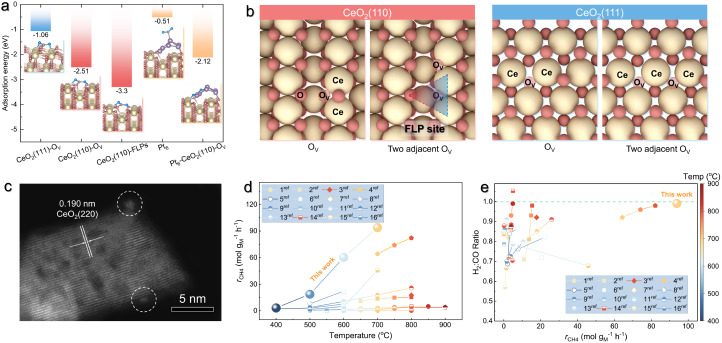
Theoretical investigation and catalytic performance. (a) The CO_2_ adsorption behaviors on the CeO_2_(110) surface with various active sites. (b) Optimized structure of one O_V_ and two adjacent O_V_s on CeO_2_(110) and CeO_2_(111) surfaces. (c) HAADF-STEM image of Pt_cluster_/CeO_2_-FLP. (d) Comparison of CH_4_ conversion rates over Pt_cluster_/CeO_2_-FLP and other state-of-the-art catalysts (see Table S2 for more details). (e) Comparison of CH_4_ conversion rates and H_2_ : CO ratios over Pt_cluster_/CeO_2_-FLP and other state-of-the-art catalysts under similar reaction conditions (see Table S2 for more details).

Density functional theory (DFT) calculations were employed to guide this design. As shown in Fig. 2a and S1–S5, CO_2_ adsorption at the oxygen vacancy (O_V_) on CeO_2_(110) (CeO_2_(110)-O_V_) is significantly stronger than that on CeO_2_(111)-O_V_, with an adsorption energy of −2.51 eV. Although this value is more negative than that for CO_2_ adsorption on Pt_6_ clusters, it remains comparable to that at the Pt_6_–CeO_2_ interface, indicating that an isolated O_V_ is insufficient to achieve the full spatial decoupling. Notably, constructing adjacent O_V_ pairs on CeO_2_(110) creates the FLP sites (Ce^3+^…Ce^3+^, O^2−^; Fig. 2b), which dramatically enhance CO_2_ adsorption, with an energy of −3.30 eV (Fig. 2a). In contrast, creating more O_V_s on CeO_2_(111) merely increases the number of O_V_s without creating a distinct adsorption site (Fig. 2b). These results confirm that a FLP on CeO_2_(110), coupled with Pt clusters, provides an ideal platform for spatially decoupled catalysis toward efficient and stoichiometric DRM.

Guided by the above analysis, we synthesized porous CeO_2_ nanorods (denoted as CeO_2_-FLP) designed to host FLP sites through a sequential low- and high-pressure hydrothermal method.^21,32^ Dark-field transmission electron microscopy (TEM) images revealed a well-defined porous architecture with an average pore size of 2–3 nm (Fig. S6a). High-resolution TEM showed lattice fringes with a lattice fringe spacing of 0.19 nm, corresponding to the CeO_2_ [220] planes (Fig. S6b), indicating the preferential growth along the [110] direction. X-ray photoelectron spectroscopy (XPS) analysis of Ce 3d and O 1s peaks indicated the abundance of surface defects in CeO_2_-FLP, as evidenced by the Ce^3+^ (29.4%, Fig. S7a) and Ce^3+^-O (52.4%, Fig. S7b) fractions. The abundance of O_V_, along with the exposure of (110) facets, suggests the effective formation of FLP sites on the CeO_2_-FLP surface.^21,33,34^

Subsequently, Pt clusters were deposited on CeO_2_-FLP (Pt_cluster_/CeO_2_-FLP) using H_2_PtCl_6_·6H_2_O as the precursor *via* a photo-assisted reduction process. The actual Pt loading was quantified to be 0.9 wt% using inductively coupled plasma optical emission spectroscopy (ICP-OES). High-angle annular dark-field scanning transmission electron microscopy (HAADF-STEM) images of Pt_cluster_/CeO_2_-FLP revealed brightness variation on CeO_2_-FLP (Fig. 2c), indicating the presence of Pt clusters with an average size of 0.9 ± 0.1 nm (Fig. S8). CO chemical adsorption determined a Pt dispersion of 43.2% (Table S1). Importantly, Pt_cluster_/CeO_2_-FLP retained high Ce^3+^ (30.9%) and Ce^3+^-O (55.2%) fractions (Fig. S7), confirming the preservation of the FLP-rich surface.

The DRM performances of Pt_cluster_/CeO_2_-FLP were evaluated in a fixed-bed reactor at a feed ratio of CH_4_ : CO_2_ : N_2_ (2 : 2 : 1). At 700 °C, Pt_cluster_/CeO_2_-FLP achieved a CH_4_ conversion of 75.7% (Fig. S9), approaching the thermodynamic equilibrium (76.0%, at 700 °C), along with a CH_4_ conversion rate of 93.9 mol g_Pt_^−1^ h^−1^. This activity substantially exceeds that of state-of-the-art catalysts (Fig. 2d and Table S2). Importantly, the H_2_ and CO production rates reached 187.7 mol g_Pt_^−1^ h^−1^ and 189.5 mol g_Pt_^−1^ h^−1^, respectively, giving a near-stoichiometric H_2_ : CO ratio of 0.99. Therefore, Pt_cluster_/CeO_2_-FLP delivers an unprecedented combination of high activity and ideal product stoichiometry for DRM (Fig. 2e and Table S2).

### Critical functions of the FLP sites

**Fig. 3:**
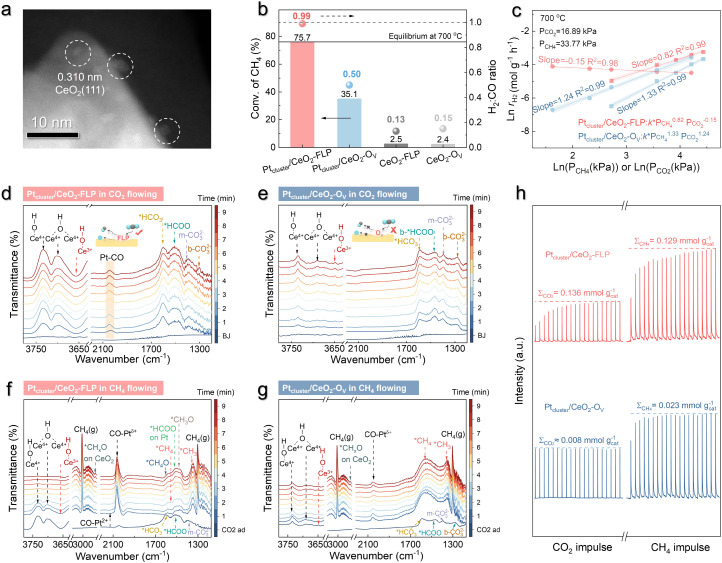
Investigations on the critical functions of FLP. (a) HAADF-STEM image of Pt_cluster_/CeO_2_-O_V_. (b) CH_4_ conversions and H_2_/CO ratios at 700 °C with a WHSV of 30 000 mL g_cat_^−1^ h^−1^. (c) Kinetic measurements of Pt_cluster_/CeO_2_-FLP and Pt_cluster_/CeO_2_-O_V_. *In situ* DRIFTS spectra of (d) Pt_cluster_/CeO_2_-FLP and (e) Pt_cluster_/CeO_2_-O_V_ under CO_2_ flow. *In situ* DRIFTS spectra of (f) Pt_cluster_/CeO_2_-FLP and (g) Pt_cluster_/CeO_2_-O_V_ under CH_4_ flow after CO_2_ flow. Note: all of the catalysts were pre-treated by Ar flow for 30 min. Initially, a flow of 50 vol.% CO_2_/Ar was introduced and the DRIFTS signals were collected at 350 °C every 1 min. Subsequently, the 50 vol.% CO_2_/Ar gas was switched to a flow of 50 vol.% CH_4_/Ar. The DRIFTS signals were further collected at 350 °C every 1 min. (h) Pulsed reactions of CO_2_ and CH_4_ over Pt_cluster_/CeO_2_-FLP and Pt_cluster_/CeO_2_-O_V_.

As a catalyst design concept (Fig. 1c), the FLP sites on the CeO_2_(110) surface, rather than Pt clusters or Pt–CeO_2_ interfaces, are responsible for the strong CO_2_ adsorption and activation. This capability is absent on the CeO_2_(111) facet, theoretically leading to inferior DRM performance. We synthesized nano-octahedral CeO_2_ (denoted as CeO_2_-O_V_) exclusively exposing (111) facets, which are structurally incompatible with FLP formation and thus possess only conventional O_V_ (Fig. 2b and S10).^21^ Consistent with this morphology, CeO_2_-O_V_ exhibited a lower concentration of surface defects, with Ce^3+^ and Ce^3+^-O_V_ fractions of 19.3% and 40.3%, respectively (Fig. S11 and Table S1). Using the same photo-assisted deposition method, Pt clusters were loaded onto CeO_2_-O_V_ (Pt_cluster_/CeO_2_-O_V_) at 1.1 wt% loading. HAADF-STEM image confirmed the Pt clusters with an average size of 0.9 ± 0.3 nm (Fig. 3a and S12). Meanwhile, Pt deposition did not substantially alter the surface defect of CeO_2_-O_V_, with Ce^3+^ and Ce^3+^-O fractions remaining at 20.4% and 42.4%, respectively (Fig. S11 and Table S1). Under identical reaction conditions, Pt_cluster_/CeO_2_-O_V_, despite possessing comparable Pt clusters, exhibited markedly inferior DRM performance. Specifically, CH_4_ conversion was 35.1% (Fig. 3b), 2.2-fold lower than that of Pt_cluster_/CeO_2_-FLP (75.7%) at 700 °C. Meanwhile, the H_2_ : CO ratio plummeted to 0.5, indicating severe deviation from stoichiometric product ratios (Fig. 3b). These comparative results unequivocally establish that the FLP sites are essential for achieving exceptional DRM activity and stoichiometry.

Kinetic analysis at low conversions (<20%) further elucidates the critical role of FLP. The CO_2_ reaction order over Pt_cluster_/CeO_2_-FLP was significantly lower than that over Pt_cluster_/CeO_2_-O_V_ (Fig. 3c), indicating higher CO_2_ surface coverage on the FLP-containing catalyst. Arrhenius plots (ln *k vs.* 1/*T*, Fig S13 and S14) revealed that the activation energy (*E*_a_) for CO_2_ conversion on CeO_2_-FLP (59.6 kJ mol^−1^) was substantially lower than that on CeO_2_-O_V_ (79.1 kJ mol^−1^). Thus, the higher CO_2_ coverage on Pt_cluster_/CeO_2_ results in the stronger conversion capacity, directly distinguishing FLP from O_V_s. This trend was further supported by CO_2_ temperature-programmed desorption (CO_2_-TPD, Fig. S15), in which CeO_2_-FLP displayed an additional strong desorption peak at ∼500 °C, confirming enhanced CO_2_ adsorption at the FLP sites.

More importantly, introducing Pt clusters onto CeO_2_-O_V_ significantly reduced the *E*_a_ for CO_2_ conversion from 79.1 kJ mol^−1^ (CeO_2_-O_V_) to 71.5 kJ mol^−1^ (Pt_cluster_/CeO_2_-O_V_, Fig. S14). In the absence of FLP sites on CeO_2_ supports, CO_2_ adsorption and activation are influenced by Pt or the Pt–CeO_2_ interface, consistent with previous reports. Conversely, depositing Pt onto CeO_2_-FLP exhibited a negligible effect on the *E*_a_ for CO_2_ conversion, as evidenced by the nearly identical values for Pt_cluster_/CeO_2_-FLP (55.7 kJ mol^−1^) and CeO_2_-FLP (59.6 kJ mol^−1^; Fig. S14). Different form conventional O_V_ sites, these kinetic results experimentally demonstrate that CO_2_ activation occurs predominantly on FLP sites rather than at Pt clusters or the Pt–O–Ce interfaces, with Pt clusters playing a negligible direct role in CO_2_ transformation.

Subsequently, *in situ* diffuse reflectance infrared Fourier transform spectroscopy (DRIFTS) was employed to monitor the roles of FLP under the given reaction conditions. Both Pt_cluster_/CeO_2_-FLP (Fig. 3d) and Pt_cluster_/CeO_2_-O_V_ (Fig. 3e) exhibited characteristic vibrational signatures of bicarbonate (*HCO_3_, at 1602 cm^−1^) and formate (*HCOO, 1505 cm^−1^) intermediates, confirming the CO_2_ chemisorption on CeO_2_ supports.^35,36^ However, Pt_cluster_/CeO_2_-FLP exhibited strong capacity of transformation of b-CO_3_^2−^, as evidenced from their much weaker adsorption peaks at ∼1300 cm^−1^. Furthermore, Pt_cluster_/CeO_2_-FLP displayed an obvious *CO adsorption band (∼2046 cm^−1^) associated with Pt clusters, which was not detected on Pt_cluster_/CeO_2_-O_V_. This critical distinction confirms that CeO_2_-FLP enables a direct CO_2_-to-CO reduction pathway.^37,38^ Moreover, during CO_2_ exposure on Pt_cluster_/CeO_2_-FLP, the Ce^3+^-OH stretching mode (∼3656 cm^−1^) attenuated while Ce^4+^-OH features (∼3733 cm^−1^ and ∼3698 cm^−1^) intensified (Fig. 3d and e),^39–41^ indicating the accumulation of *O species within the CeO_2_-FLP lattice during the CO_2_-to-CO reduction, accompanied by the Ce^3+^-to-Ce^4+^ oxidation.

When a CH_4_ flow was introduced into the *in situ* reactor, Pt_cluster_/CeO_2_-FLP exhibited a significantly enhanced intensity of *CO adsorption on Pt clusters, indicating that the CH_4_ oxidation occurred on the Pt sites (Fig. 3f). Meanwhile, the vibrational features corresponding to the *CH_3_ (∼1342 cm^−1^), *CH_3_O (∼1471 cm^−1^), *CH_4_O (∼1583 cm^−1^) and Pt-bound *HCOO (∼1517 cm^−1^) species emerged during CH_4_ flow, along with the disappearance of CeO_2_-bound *HCOO bonding (∼1509 cm^−1^) and *CO_3_^2−^ (∼1397 cm^−1^) intermediates. Simultaneously, the Ce^4+^ species (Ce^4+^-OH, 3733 cm^−1^) was reduced to Ce^3+^ (Ce^3+^-OH, 3658 cm^−1^) along with these transformations. Thus, these findings collectively demonstrate that CH_4_ oxidation is mediated by lattice *O species originating from CeO_2_-FLP supports.

In contrast, Pt_cluster_/CeO_2_-O_V_ exhibited negligible activity for this reaction pathway (Fig. 3g). The CH_4_ exposure on Pt_cluster_/CeO_2_-O_V_ induced the rapid emergence of *CH_3_ (at ∼1342 cm^−1^) and *CH_4_ (at ∼1557 cm^−1^) species, indicating that CH_4_ adsorption and activation occurred on Pt sites.^42,43^ However, CO production remained consistently low, demonstrating limited further oxidation of activated *CH_3_ and *CH_4_ species to *CO. Furthermore, the partial retention of Ce^4+^ species (Ce^4+^-OH, 3733 cm^−1^) indicated the restricted *O migration during CH_4_ flow, owing to the poor capacity for CO_2_ activation/reduction of CeO_2_-O_V_. Concomitantly, the disappearance of *HCO_3_, *HCOO, and b-CO_3_^2-^ species could be attributed to the *H spillover from Pt into the CeO_2_-O_V_ supports.

Sequential CO_2_/CH_4_ pulse experiments further quantified the FLP functions. Pt_cluster_/CeO_2_-FLP showed distinct CO_2_ consumption over 14 pulse cycles, corresponding to an *O storage capacity of 0.136 mmol g_cat_^−1^. This observation contrasted sharply with Pt_cluster_/CeO_2_-O_V_, which exhibited a negligible *O storage capacity (0.008 mmol g_cat_^−1^), consistent with *in situ* DRIFTS spectra confirming only adsorption of CO_2_ on CeO_2_-O_V_ (Fig. 3h). Subsequent CH_4_ pulses revealed drastically higher consumption on Pt_cluster_/CeO_2_-FLP (0.129 mmol_CH4_ g_cat_^−1^) than that on Pt_cluster_/CeO_2_-O_V_ (0.023 mmol_CH_4__ g_cat_^−1^). The close match between CH_4_ consumption and *O storage on Pt_cluster_/CeO_2_-FLP confirms efficient transfer of the FLP-generated *O species to Pt sites for CH_4_ oxidation.

### Investigation into the functions of Pt clusters

Notably, the pronounced reduction in *E*_a_ for CH_4_ conversion upon introducing Pt clusters (Pt_cluster_/CeO_2_-FLP and CeO_2_-FLP, 64.1 kJ mol^−1^ and 112.6 kJ mol^—1^, respectively; Pt_cluster_/CeO_2_-O_V_ and CeO_2_-O_V_, 79.2 kJ mol^−1^ and 117.2 kJ mol^—1^, respectively; Fig. S14) unambiguously underscores the essential roles of Pt in CH_4_ adsorption and C–H bond activation. Moreover, the comparable CH_4_ reaction orders observed on both Pt_cluster_/CeO_2_-FLP and Pt_cluster_/CeO_2_-O_V_ indicated that CH_4_ activation kinetics were weakly influenced by CeO_2_ (Fig. 3c). Taken together, the higher reaction orders and activation energies associated with CH_4_ conversion indicated that CH_4_ activation constitutes the rate-determining step in the DRM reaction over Pt_cluster_/CeO_2_-FLP, particularly under preconditions where CO_2_ is efficiently activated by FLP.

To further decipher the mechanistic roles of Pt species in CH_4_ activation, the single-atom Pt and Pt nanoparticles (average size 2.2 ± 0.1 nm) were deposited on CeO_2_-FLP, yielding Pt_1_/CeO_2_-FLP (Fig. S16) and Pt_NP_/CeO_2_-FLP (Fig. S17), respectively. ICP-OES determined Pt loadings of 0.5 wt% for Pt_1_/CeO_2_-FLP and 1.0 wt% for Pt_NP_/CeO_2_-FLP. Notably, the surface properties of the supports in Pt_1_/CeO_2_-FLP and Pt_NP_/CeO_2_-FLP were similar to those of Pt_cluster_/CeO_2_-FLP (Fig. S18 and Table S1), enabling the systematic investigations of Pt speciation effects on CH_4_ activation while excluding the influences of supports.

**Fig. 4:**
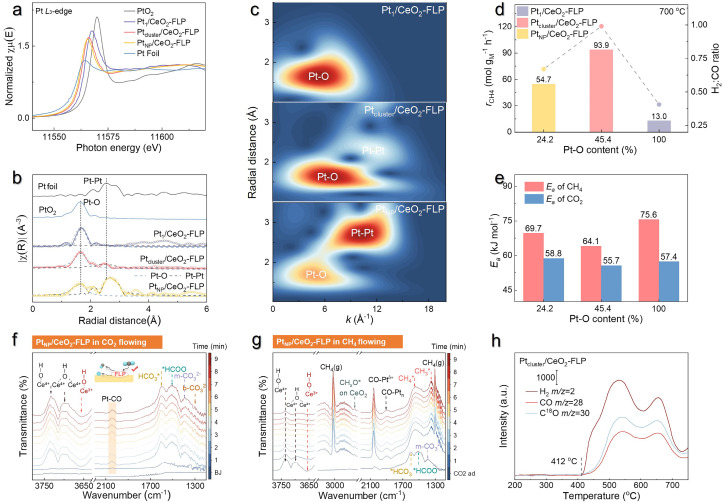
Investigation on the functions of Pt clusters. (a) XANES spectra of Pt foil, Pt_cluster_/CeO_2_-FLP, Pt_1_/CeO_2_-FLP, Pt_NP_/CeO_2_-FLP and PtO_2_. (b) The *k*^3^ weighted Fourier-transformed spectra derived from the EXAFS spectra. (c) Wavelet-transform plots by using the *k*^3^ space. (d) Direct correlation between Pt–O bond contents and CH_4_ conversion rates/H_2_ : CO ratios. (e) Plots of Pt–O bond contents *vs.* calculated *E*_a_ values of Pt_cluster_/CeO_2_-FLP, Pt_1_/CeO_2_-FLP, and Pt_NP_/CeO_2_-FLP for DRM. *In situ* DRIFTS spectra of Pt_NP_/CeO_2_-FLP under (f) CO_2_ flow and (g) CH_4_ flow after CO_2_ flow. Note: all of the catalysts were pre-treated by a flow of Ar for 30 min. Then, a flow of 50 vol.% CO_2_/Ar was introduced and the DRIFTS signals were collected at 350 °C every 1 min. Subsequently, the 50 vol.% CO_2_/Ar gas was switched to a flow of 50 vol.% CH_4_/Ar. The DRIFTS signals were further collected at 350 °C every 1 min. (h) *In situ* mass spectrometry analysis of the temperature-programmed CH_4_ oxidation of Pt_cluster_/CeO_2_-FLP upon pre-treatment with H_2_^18^O.

The Pt L_3_ edge X-ray absorption near-edge structure (XANES) was used to investigate the electronic states of Pt. The white line peak of Pt_1_/CeO_2_-FLP was located at 11 567.4 eV, close to that of PtO_2_ (Fig. 4a). The *k*^3^-weight Fourier transforms of extended X-ray absorption fine structure (EXAFS) spectra of Pt_1_/CeO_2_-FLP delivered one prominent peak at ∼1.64 Å, which was labeled as the Pt–O bond (Fig. 4b and S19). The lack of Pt–Pt coordination confirmed the atomically dispersed Pt supported on CeO_2_-FLP. The wavelet transform analysis directly revealed the absence of Pt–Pt bonds in Pt_1_/CeO_2_-FLP (Fig. 4c). In contrast, Pt_cluster_/CeO_2_-FLP and Pt_NP_/CeO_2_-FLP exhibited the white line peaks between Pt foil and PtO_2_ (Fig. 4a), indicating the mixed valence states of Pt. Meanwhile, both Pt–Pt and Pt–O bonds were clearly observed from the *k*^3^-weight Fourier transforms of EXAFS spectra. Compared to Pt_NP_/CeO_2_-FLP, the higher white line peak revealed more amount of the Pt–O bond in Pt_cluster_/CeO_2_-FLP, which could be clearly observed from the wavelet transform analysis. Quantitatively, Pt_1_/CeO_2_-FLP, Pt_cluster_/CeO_2_-FLP and Pt_NP_/CeO_2_-FLP exhibited the Pt–O fractions of 100%, 45.4% and 24.2%, respectively, as well as the Pt–Pt fractions of 0%, 54.6% and 75.8%, respectively (Table 1).

**Table 1:** Fitting results of EXAFS for Pt_1_/CeO_2_-FLP, Pt_cluster_/CeO_2_-FLP and Pt_NP_/CeO_2_-FLP^a^

Sample	Paths	*N* b	*R* (Å)^c^	*σ* ^2^ (×10^−3^ Å^2^)^d^	*S*_0_^2^	*E*_0_ (eV)	*R* Factor
Pt foil	Pt–Pt	12.0	2.77	—	—	—	—
PtO_2_	Pt–O	6.0	2.07	—	—	—	—
Pt_1_/CeO_2_-FLP	Pt–O	3.4 ± 0.1	2.04 ± 0.07	5.2 ± 0.9	0.8	7.4	0.018
Pt_cluster_/CeO_2_-FLP	Pt–O	4.4 ± 0.4	2.07 ± 0.02	2.1 ± 0.2	0.8	8.5	0.012
	Pt–Pt	5.3 ± 0.5	2.82 ± 0.16	5.2 ± 0.1			
Pt_NP_/CeO_2_-FLP	Pt–O	3.0 ± 0.7	2.10 ± 0.03	7.0 ± 2.9	0.8	8.7	0.008
	Pt–Pt	9.4 ± 0.2	2.78 ± 0.14	5.8 ± 0.6			

a The data ranges used in the fit are 2.0 ≤ *k* ≤ 13.0 Å^−1^ and 1.2 ≤ *R* ≤ 3.5 Å, and depend on the quality of data. The number of fitted variable parameters was 10, which was smaller than the total number of independent data points, approximately 16.1. *R*-Factors for these fittings are all below 0.02.

b Average coordination number. The half path length.

c The paths for Pt–O, Pt–Pt are from the crystal structure of PtO_2_ (*P*6_3_*mc*) and Pt (*Fm*3̄*m*).

d Debye–Waller factor.

For the DRM reaction, Pt_cluster_/CeO_2_-FLP, featuring the co-existence of Pt–O and Pt–Pt bonds, delivered the highest CH_4_ conversion rate compared to Pt_1_/CeO_2_-FLP and Pt_NP_/CeO_2_-FLP (Fig. 4d and S20), as well as the lowest *E*_a_ of 64.1 kJ mol^−1^ for CH_4_ conversion (Fig. 4e and S21). These observations confirm that the highest intrinsic activity of Pt clusters originates from a synergistic interplay between Pt–Pt and Pt–O bonds in CH_4_ activation. This synergy arises from the presence of coordinatively unsaturated metal and oxygen on the Pt surface, which promotes the formation of the adsorbed CH_4_ σ-complexes and then facilitates C–H bond cleavage in the complexes.^25,44^ Furthermore, due to the similar surface properties of supports, all Pt/CeO_2_-FLP catalysts exhibited comparable *E*_a_ values for CO_2_ conversion (Fig. 4e), further confirming the strong capacity of FLP for CO_2_ adsorption/activation and the negligible influence of Pt active sites on this process.

The CH_4_ activation on Pt/CeO_2_-FLP was further investigated by CH_4_ temperature-programmed reduction (CH_4_-TPR). After the pre-treatment with Ar purging, a CH_4_ flow was introduced to probe the catalyst surface. The CeO_2_-FLP supports alone exhibited no detectable activation peaks (Fig. S22), further confirming that the CH_4_ adsorption and activation occur exclusively at Pt sites. Importantly, Pt_cluster_/CeO_2_-FLP exhibited the strongest peak intensity and the lowest initiation temperature compared to both Pt_1_/CeO_2_-FLP and Pt_NP_/CeO_2_-FLP (Fig. S22). These observations directly confirmed the highest capacity of Pt_cluster_/CeO_2_-FLP for the CH_4_ adsorption and activation in DRM.

Subsequently, *in situ* DRIFTS experiments were performed to compare the behaviors of Pt clusters and nanoparticles under the given reaction conditions. Under CO_2_ flow, Pt_NP_/CeO_2_-FLP exhibited spectral changes nearly identical to those of Pt_cluster_/CeO_2_-FLP, consistent with their similar CeO_2_ surface properties for CO_2_ adsorption (Fig. 4f). Upon switching to CH_4_ flow, characteristic peaks corresponding to adsorbed *CH_4_ (1557 cm^−1^) appeared on Pt_NP_/CeO_2_-FLP, confirming CH_4_ adsorption (Fig. 4g). Unlike Pt_cluster_/CeO_2_-FLP, no clear signals for *CH_*x*_O intermediates were detected on Pt_NP_/CeO_2_-FLP. Moreover, the Ce^4+^-OH peak persisted even after 9 min CH_4_ flow, indicating restricted *O migration from the support to the Pt nanoparticles. Together with CH_4_-TPR results, these observations demonstrate that Pt clusters uniquely promote the migration of *O species, which in turn enhances CH_4_ activation and overall DRM activity.

Building on the foregoing analysis, the spatially separated Pt clusters and FLP sites serve distinct roles in CH_4_ oxidation and CO_2_ reduction, respectively, with *O migration acting as the key coupling step, as illustrated in Fig. 1c. To further probe the origin and transfer of *O species, we performed ^18^O-isotope labeling experiments. Because labeled CO_2_ can lead to ambiguity from residual adsorption or desorption of ^18^O-containing species, we designed the experiment using H_2_^18^O pretreatment, which readily dissociates on CeO_2_ but not on Pt clusters.^32,45,46^ Specifically, after pretreating Pt_cluster_/CeO_2_-FLP with H_2_^18^O at 150 °C for 30 min, purging with Ar for 30 min was performed to remove physically adsorbed species. The programmed temperature increase of CH_4_ oxidation from 200 °C to 750 °C (10 vol.% CH_4_/He, 50 mL min^−1^) revealed the appearance of the C^18^O/CO signal (Fig. 4h), which directly demonstrated that *O species stored in the CeO_2_-FLP support participate in CH_4_ oxidation through oxygen transfer to Pt clusters. Furthermore, the H_2_ signal appears simultaneously with the CO/C^18^O signals, indicating that CH_4_ dissociates on Pt sites, with CO and H_2_ formation. Notably, the observed C^18^O signal indicates that the oxygen involved in CH_4_ oxidation originated predominantly from H_2_^18^O-derived *^18^O species stored in the CeO_2_-FLP support rather than from the lattice oxygen. Together, these results strongly support a spatially decoupled reaction pathway in which *O migration couples CO_2_ reduction on the support with CH_4_ oxidation on Pt clusters.

### Activity matching between Pt clusters and FLP sites

**Fig. 5:**
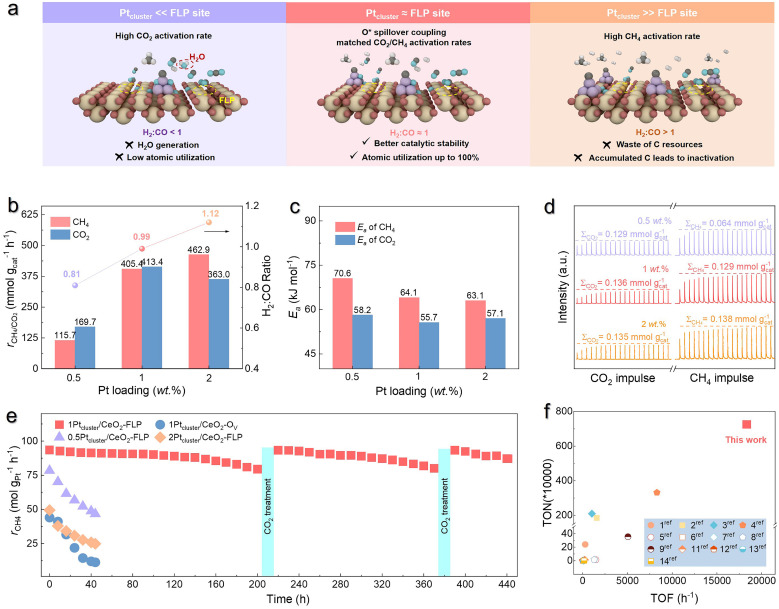
Activity matching. (a) Scheme of activity matching between Pt clusters and FLPs for the DRM reaction. (b) Influences of Pt loadings on the CH_4_/CO_2_ generation rates and H_2_ : CO ratios of the Pt_cluster_/CeO_2_-FLP catalysts for DRM. (c) Influence of Pt loadings on the *E*_a_ values of Pt_cluster_/CeO_2_-FLP for CO_2_ and CH_4_ conversions. (d) Pulsed reactions of CO_2_ and CH_4_ over Pt_cluster_/CeO_2_-FLP with various Pt loadings. (e) Catalytic stability of various catalysts with a WHSV of 30 000 mL g_cat_^−1^ h^−1^ (CH_4_ : CO_2_ : N_2_ = 2 : 2 : 1) at 700 °C. CO_2_ treatment conditions: CO_2_/N_2_ at 700 °C for 10 h with a WHSV of 18 000 mL g_cat_^−1^ h^−1^. (f) Comparison of TON and TOF between Pt_cluster_/CeO_2_-FLP and other state-of-the-art catalysts under similar reaction conditions (see Table S2 for more details).

Achieving activity balance between the spatially decoupled CO_2_ reduction and CH_4_ oxidation is critical for high-performance DRM under the *O migration coupling process, as it ensures near stoichiometric H_2_ : CO ratios (1 : 1) and long-term catalytic stability. As shown in Fig. 5a, this activity balance is inherently determined by the quantitative relationship between FLP sites (responsible for CO_2_ reduction) and Pt clusters (mediating CH_4_ oxidation). While the FLP sites remain fixed within the CeO_2_-FLP supports, we precisely adjusted the number of Pt centers by controlling the Pt loadings. HAADF-STEM images revealed that increasing Pt loadings from 0.5 wt% to 2.0 wt% enhanced the densities of Pt clusters while preserving their similar cluster dimensions (an average diameter of 0.9–1.0 nm, Fig. S23). This controlled variation in Pt cluster population enabled systematic modulation of the FLP-to-Pt site ratios, a crucial factor governing overall DRM performance.

For DRM, Pt loading significantly influenced reaction kinetics and product stoichiometry. At 0.5 wt% Pt loading, the CH_4_ conversion rate of 115.7 mmol g_cat_^−1^ h^−1^ was lower than the CO_2_ conversion rate of 169.7 mmol g_cat_^−1^ h^−1^, resulting in a significantly reduced H_2_ : CO ratio of 0.81 at 700 °C (Fig. 5b and S24). Elevating the Pt loading to 1 wt%, accompanied with a higher density of Pt clusters, drastically improved the enhanced CH_4_ conversion rate (405.4 mmol g_cat_^−1^ h^−1^), matching the CO_2_ conversion rate of 405.6 mmol g_cat_^−1^ h^−1^. Simultaneously, the H_2_ : CO ratio approached 0.99, nearly achieving a stoichiometric ratio of 1 (Fig. 5b). Therefore, increasing the Pt loading facilitated the optimal matching between Pt clusters and FLP sites.

Further increasing the Pt loading to 2 wt% resulted in CH_4_ and CO_2_ conversion rates similar to those observed for Pt_cluster_/CeO_2_-FLP with 1 wt% Pt loading (Fig. 5b and S24). This observation demonstrated that the CH_4_ activation and conversion depended on the migration of *O species generated from the CO_2_ reduction on FLP of CeO_2_-FLP, rather than the availability of Pt clusters. Consequently, CH_4_ conversion rates plateau at the 1 wt% Pt loading, as additional Pt clusters cannot compensate for the limited *O supply from the FLP. The extra CH_4_ conversion would lead to carbon deposition on the catalyst surface (Fig. 5a). Carbon balance analysis before and after the reaction revealed greater carbon imbalance for Pt_cluster_/CeO_2_-FLP with higher Pt loadings, indicating direct carbonization of CH_4_ on Pt (Fig. S25). Raman spectroscopy of the spent Pt_cluster_/CeO_2_-FLP catalysts proved the direct evidence of carbonaceous species (Fig. S26). After 45 h reaction at 700 °C, thermogravimetric analysis (TGA) quantified a carbon deposition rate of 2.69 mg g_cat_^−1^ h^−1^ for the 2 wt% Pt_cluster_/CeO_2_-FLP catalysts, which was ∼7 times higher than that for 1 wt% and 0.5 wt% catalysts (0.39 and 0.38 mg g_cat_^−1^ h^−1^, respectively; Fig. S27). Collectively, these results reveal the critical role of the balanced FLP-to-Pt site ratios in achieving efficient DRM performance with stoichiometric H_2_ : CO output.

Kinetic analysis further clarified the activity matching requirements between Pt clusters and FLP sites (Fig. S28). The comparable *E*_a_ values of Pt_cluster_/CeO_2_-FLP for CO_2_ conversion indicated that CO_2_ adsorption and transformation predominantly occurred on FLP sites (Fig. 5c). Theoretically, similar Pt clusters in Pt_cluster_/CeO_2_-FLP should yield comparable *E*_a_ values for CH_4_ conversion. However, when the Pt loading was 0.5 wt%, the relatively large distance between Pt clusters and FLP sites resulted in a higher *E*_a_ value for CH_4_ conversion. In contrast, sufficient Pt loading (>1.0 wt%) reduces the average spatial distance between Pt clusters and FLP sites, leading to sufficient migration and supply of *O species and thus delivering comparable *E*_a_ values for CH_4_ conversion. When the number of Pt clusters matched that of FLP sites, the *E*_a_ values for CH_4_ conversion and CO_2_ conversion were also comparable, thereby facilitating the DRM reaction with a near-stoichiometric ratio of H_2_ : CO ≈ 1. This kinetic deconvolution highlights the critical roles of the available Pt clusters and FLP sites in balancing *O supply (from FLPs) and CH_4_ activation (at Pt) for DRM.

The CO_2_ pulse experiments revealed that Pt_cluster_/CeO_2_-FLP with varying Pt loadings exhibited comparable *O storage capacities (0.129–0.136 mmol g_cat_^−1^, Fig. 5d). These findings further confirm that FLP sites act as active centers for CO_2_ reduction, generating reactive *O species that subsequently participate in CH_4_ oxidation pathways. Specially, the Pt loading of 0.5 wt% yielded a low rate of 0.043 mmol g_cat_^−1^ due to the insufficient amount and density of Pt clusters. When the Pt loadings were elevated to 1.0 wt% and 2.0 wt%, the CH_4_ consumption increased to 0.129 mmol g_cat_^−1^ and 0.138 mmol g_cat_^−1^, respectively. Excessive Pt loading did not enhance DRM activity, as the FLP sites imposed a kinetic bottleneck of the *O supply from the CO_2_ reduction.

Notably, the observed CH_4_ consumption over Pt_cluster_/CeO_2_-FLP did not scale with the population of Pt clusters as the Pt loading increased from 1.0 wt% to 2.0 wt%, contradicting the scenario where only interfacial oxygen is involved in the reaction. This deviation indicates that the *O species are not confined to the Pt–CeO_2_ interface. Instead, the facile migration of oxygen across the CeO_2_-FLP support enables the entire oxygen reservoir of the carrier to participate in CH_4_ oxidation. Consequently, these findings further demonstrate that CO_2_ activation occurs extensively on the FLP sites of CeO_2_-FLP, thereby achieving spatial decoupling from CH_4_ oxidation at the Pt clusters.

Additionally, the optimal synergy of Pt clusters and FLP sites directly enhances catalytic stability. Pt_cluster_/CeO_2_-O_V_, lacking FLP sites, exhibited inferior stability, as evidenced by the substantially declined conversions of CH_4_ and CO_2_ during a period of 45 h (Fig. 5e). Similarly, Pt_cluster_/CeO_2_-FLP with either insufficient (0.5 wt%) or excessive (2.0 wt%) Pt loading exhibited compromised durability, attributable respectively to limited CH_4_ activation capacity and inadequate *O-migration kinetics. In contrast, the optimally matched Pt_cluster_/CeO_2_-FLP (1 wt% Pt loading) catalyst delivered exceptional long-term stability, maintaining nearly constant CH_4_ conversion rates (Fig. 5e) and a near-stoichiometric H_2_ : CO ratio (Fig. S29) for over 400 h. During the initial 100 h, when the H_2_/CO ratio remained near 1, no detectable water appeared in the product stream, indicating effective suppression of the RWGS reaction. However, XPS analysis of the spent Pt_cluster_/CeO_2_-FLP catalyst revealed both reduced surface defects and accumulated carbonaceous species (Fig. S30), which together mask Pt clusters and FLP sites. Notably, a slight 15% loss in activity during long-term testing was completely reversed by a simple CO_2_ treatment (Fig. 5e). This remarkable stability reflected in a turnover number (TON) exceeding 7 200 000 per exposed Pt site for CH_4_ conversion (Fig. 5f and S31). To the best of our knowledge, Pt_cluster_/CeO_2_-FLP represents the first to simultaneously achieve such a record-high TON and unprecedented activity, with its TON value nearly double the previously reported maximum (Fig. 5f).

Finally, we propose a catalytic process for DRM on Pt_cluster_/CeO_2_-FLP that spatially decouples CO_2_ reduction and CH_4_ oxidation, as illustrated in Fig. 1c. The CO_2_ reduction step follows a Mars–van Krevelen (MvK) mechanism: CO_2_ is adsorbed and activated at FLP sites, directly forming CO and leaving *O species on the CeO_2_-FLP support. Subsequently, the *O species migrate from FLP sites to Pt clusters. On Pt clusters, CH_4_ is converted to CO and H_2_*via* *CH_*x*_O intermediates, facilitated by the migrated *O species. Thus, while the individual CO_2_ reduction step obeys the classical MvK redox cycle, the overall process represents a modified MvK-type pathway enabled by spatial decoupling and *O migration between distinct active sites. This design separates CO_2_ reduction (FLP sites) from CH_4_ oxidation (Pt clusters) and couples them through oxygen spillover. Through precise matching of Pt clusters and FLP sites, the two half-reactions are efficiently coupled, leading to a near-stoichiometric H_2_/CO ratio and sustained DRM activity.

## Conclusions

In summary, this work demonstrates that *O migration across a spatially decoupled Pt/CeO_2_ catalyst enables near-stoichiometric DRM under continuous operation, delivering durable and high-performance DRM catalysis with a stoichiometric H_2_ : CO ratio. This catalyst separates the antagonistic adsorption and activation steps: CO_2_ is selectively reduced at FLP sites on the CeO_2_(110) facet, while CH_4_ is partially oxidized on Pt clusters, with *O shuttling dynamically between the two sites. This *O-transport mechanism intrinsically couples the two half-reactions, maintains a balanced redox cycle, and effectively maintains a H_2_ : CO ratio of 0.99 while suppressing coke formation and deactivation pathways. As a result, the catalyst delivers a record CH_4_ conversion rate of 93.9 mol g_Pt_^−1^ h^−1^ at 700 °C with stable operation exceeding 400 h under continuous, isothermal conditions. Although the present study employs Pt/CeO_2_(110) as a model platform, the underlying principle is not limited to this specific combination. The key requirements are: (i) a reducible oxide support capable of forming FLP sites (adjacent oxygen vacancies) that strongly activate CO_2_ and (ii) metal sites (clusters or nanoparticles) that can activate CH_4_ and accept migrating *O species. Many earth-abundant metals (*e.g.*, Ni, Co, and Ru) and other reducible oxides (*e.g.*, TiO_2_, WO_3_, and In_2_O_3_) are known to exhibit similar oxygen spillover behavior and have been reported to form frustrated Lewis pair-like defects under reducing conditions. Therefore, we anticipate that the spatial decoupling strategy can be extended to more practical catalyst compositions, guided by the design principles established here.

## Author contributions

W. L. performed most of the experiments. J. Y., W. G. and Q. G. participated in data analysis. S. Z. and Y. Q. designed the studies and wrote the paper. All authors discussed the results and commented on the manuscript.

## Conflicts of interest

There are no conflicts to declare.

## Supplementary Material

SC-017-D6SC03014A-s001

## Data Availability

The data supporting the findings of this study are available in the main text and supplementary information (SI). Supplementary information: experimental details, catalyst synthesis procedures, characterization data, catalytic performance data, kinetic analysis, *in situ* spectroscopic results, computational details, figures and tables. Additional raw data are available from the corresponding author upon reasonable request. See DOI: https://doi.org/10.1039/d6sc03014a.
