# Supplementary material for: Spatial decoupling of CH4 oxidation and CO2 reduction enables near-stoichiometric dry reforming of methane
Source: Chem Sci. 2026 Jun 1;17(28):13952–61. doi: 10.1039/d6sc03014a (PMC13251374; doi:10.1039/d6sc03014a)
Supplement: SC-017-D6SC03014A-s001 [file SC-017-D6SC03014A-s001.pdf]

Supplementary Information (SI) for Chemical Science.
This journal is © The Royal Society of Chemistry 2026

Supplementary information

Spatial decoupling of CH_4 oxidation and CO_2 reduction enables near-stoichiometric dry reforming of methane

Wenbin Li[a], Jiyun Ren[a], Wenjie Guo[a], Qing Guo[a], Sai Zhang[a,*] and Yongquan Qu[a,*]

[a] *School of Chemistry and Chemical Engineering, Northwestern Polytechnical University, Xi'an, 710072, China*

*Correspondence and requests for materials should be addressed to Zhang S. and Qu Y. Q. (Email: zhangsai1112@nwpu.edu.cn and yongquan@nwpu.edu.cn)

Materials

Cerium(III) nitrate hexahydrate ($Ce(NO_3)_3·6H_2O$), $H_2PtCl_6·6H_2O$, NaOH were purchased from Energy Chemical. All the reagents were of analytical grade and used directly without any further purification before the experiments.

Method

Preparation of the $Pt_{cluster}/CeO_2$-FLP catalysts

Initially, 5 mL of $Ce(NO_3)_3$ solution (0.8 mmol mL^{-1}) was added into 75 mL of NaOH solution (6.4 mmol mL^{-1}) under vigorous stirring at room temperature. After stirring for 30 min, the mixture in a 100 mL Pyrex bottle was hydrothermally treated at 100 °C for 24 h. Then, the obtained $CeO_2/Ce(OH)_3$ precursors were alternately washed by H_2O and ethanol for three times. After drying at 60 °C, the $CeO_2/Ce(OH)_3$ precursors were re-dispersed in water to give a stock solution of 2 mg mL^{-1}, which were further hydrothermally treated at 180 °C for 12 h. Finally, the CeO_2-FLP products were collected by centrifugation and dried overnight at 60 °C.

The $Pt_{cluster}/CeO_2$-FLP catalysts were synthesized by the photo-assisted deposition method. Initially, 300 mg of CeO_2-FLP was dispersed in a mixed solvent of H_2O (36 mL) and methanol (4 mL). Subsequently, 0.6 mL of an aqueous $H_2PtCl_6·6H_2O$ solution (Pt: 5 mg mL^{-1}) was added to the dispersion. The mixture was purged with Ar gas for 30 min to eliminate O_2 and ensure thorough mixing of CeO_2-FLP and H_2PtCl_6. Then, the mixture was irradiated under Xe lamp (350 W) for 4 h. During this period, the photo-induced electrons reduced the adsorbed metal precursors. Finally, the $Pt_{cluster}/CeO_2$-FLP catalysts were obtained by centrifugal separation, followed by drying at 60 °C overnight.

Catalytic performance test

The DRM reactions were carried out in a fixed-bed reactor operating at atmospheric pressure. The experimental procedure involved the loading of 50 mg of catalysts into a straight quartz tube, with temperature sensors placed both inside and outside the quartz tube. A $CH_4/CO_2/N_2$ mixture gas (40 *vol.%* CH_4, 40 *vol.%* CO_2 and 20 *vol.%* N_2) was introduced into the reactor with a total flow of 60 mL min^{-1}.

The gas products were analyzed online using a gas chromatography equipped with both TCD and FID detectors.

The conversion of CH_4 is calculated as the following:

$$\mathrm{Conv.}_{\mathrm{CH_4}}(\%)=\frac{[\mathrm{CH_4}]_{\mathrm{in}}-[\mathrm{CH_4}]_{\mathrm{out}}}{[\mathrm{CH_4}]_{\mathrm{in}}}\times 100\%$$

The conversion of CO_2 is calculated as the following:

$$\mathrm{Conv.}_{\mathrm{CO_2}}(\%)=\frac{[\mathrm{CO_2}]_{\mathrm{in}}-[\mathrm{CO_2}]_{\mathrm{out}}}{[\mathrm{CO_2}]_{\mathrm{in}}}\times 100\%$$

The H_2/CO ratio ($R_{H2/CO}$) is calculated as the following:

$$R_{H_2/\mathrm{CO}}=\frac{[\mathrm{H_2}]_{out}}{[\mathrm{CO}]_{out}}$$

Where CH_{4in}, CH_{4out}, CO_{2in}, CO_{2out}, H_{2out},and CO_{out} represent the moles of H_2, CO_2, CO and CH_4 in the effluent, respectively.

Catalytic stability test

The long-term catalytic stability of various catalysts for the DRM reaction was evaluated in a fixed-bed quartz reactor at atmospheric pressure. Typically, the catalyst was loaded into the reactor

and heated to 700 °C under an inert gas flow. After the temperature stabilized, the feed gas was switched to a mixture of $CH_4/CO_2/N_2$ = 2/2/1, corresponding to a total WHSV of 30,000 mL g_{cat}^{-1} h^{-1}. The effluent gas was continuously analyzed by online gas chromatography to monitor the CH_4 conversion, CO_2 conversion, H_2/CO ratio, and product distribution as a function of time. For catalyst regeneration, the spent catalyst was treated under a CO_2/N_2 flow at 700 °C for 10 h with a WHSV of 18,000 mL g_{cat}^{-1} h^{-1}. After CO_2 treatment, the feed gas was switched back to the DRM reaction mixture under the same reaction conditions to evaluate the recovery of catalytic activity.

Characterizations

TEM characterizations were performed on a JEOL2100F instrument with an accelerating voltage of 200 kV. The high angle annular dark-field scanning transmission electron microscopy (HAADF-STEM) and element mapping analyses were performed with a FEI Tecnai F30 microscope operated at 300 kV. XPS profiles were acquired from a Thermo Electron Model K-Alpha with Al Ka as the excitation source. The contents of Pt in various catalysts were determined by inductively coupled plasma optical emission spectrometer (ICP-OES). Alpha300R micro confocal Raman spectrometer (Raman) produced by German WITec Company was used to analyze the surface defects.

Synthesis of nano-octahedron of CeO_2 (CeO_2-O_V)

The CeO_2-O_V supports were synthesized *via* a hydrothermal method. Initially, $Ce(NO_3)_3·6H_2O$ (434.3 mg) and Na_3PO_4 (1.6 mg) were dissolved in 40 mL of H_2O. The mixture was sonicated for 30 min and transferred to a 100 mL Teflon-lined stainless-steel autoclave. The sealed autoclave was heated at 170 °C for 12 h. After cooling to room temperature, the solid product was collected by centrifugation, washed sequentially with H_2O and ethanol, then dried overnight at 60 °C in air.

Finally, the material was calcined in air at 400 °C for 4 h (ramp rate: 2 °C min^{-1}) to yield the CeO_2-O_V supports.

Preparation of the $Pt_{cluster}/CeO_2$-O_V catalysts

The $Pt_{cluster}/CeO_2$-O_V catalysts were prepared *via* a photo-deposition method. Initially, 300 mg of CeO_2-O_V was dispersed in a mixed solvent of H_2O (36 mL) and CH_3OH (4 mL). Subsequently, 0.6 mL of an aqueous $H_2PtCl_6·6H_2O$ solution (Pt: 5 mg mL^{-1}) was added to the dispersion. The resulting mixture was purged with Ar gas for 30 min to eliminate O_2 and ensure thorough mixing of CeO_2-O_V and H_2PtCl_6. Then, the mixture was irradiated under Xe lamp (350 W) for 4 h. During this period, the photo-induced electrons reduced the adsorbed metal precursors. Finally, the $Pt_{cluster}/CeO_2$-O_V catalysts were obtained by centrifugal separation, followed by drying at 60 °C overnight.

Preparation of the Pt_1/CeO_2-FLP catalysts

Initially, 300 mg of CeO_2-FLP was dispersed in a mixed solvent of H_2O (36 mL) and methanol (4 mL). Subsequently, 0.3 mL of an aqueous $H_2PtCl_6·6H_2O$ solution (Pt: 5 mg mL^{-1}) was added to the dispersion. The resulting mixture was purged with Ar gas for 30 min to eliminate O_2 and ensure thorough mixing of CeO_2-FLP and H_2PtCl_6. Then, the mixture was irradiated under Xe lamp (200 W) for 4 h. During this period, the photo-induced electrons reduced the adsorbed metal precursors. Finally, the $Pt_{cluster}/CeO_2$-FLP catalysts were obtained by centrifugal separation, followed by drying at 60 °C overnight.

Preparation of the Pt_{NP}/CeO_2-FLP catalysts

Initially, 300 mg of CeO_2-FLP was dispersed in a mixed solvent of H_2O (36 mL) and methanol (4 mL). Subsequently, 0.6 mL of an aqueous $H_2PtCl_6·6H_2O$ solution (Pt: 5 mg mL^{-1}) was added to the dispersion. The resulting mixture was purged with Ar gas for 30 min to eliminate O_2 and ensure thorough mixing of CeO_2-FLP and H_2PtCl_6. Then, the mixture was irradiated under Xe lamp (350 W)

for 4 h. During this period, the photo-induced electrons reduced the adsorbed metal precursors. The catalysts were obtained by centrifugal separation, followed by drying at 60 °C overnight. The dried powder was calcined at 350 °C under 10 *vol.%* H_2/Ar for 2 h. This was repeated three times to ensure that the Pt grows from clusters to large size particles to obtain the Pt_{NP}/CeO_2-FLP catalyst.

***In-situ* diffuse reflectance infrared Fourier transform spectroscopy (DRIFTS) measures**

In-situ DRIFTS experiments were conducted using a Thermo Nicolet Nexus FTIR spectrometer, which was equipped with a liquid nitrogen cooled HgCdTe (MCT) detector, ZnSe window and high-temperature in situ heating chamber. All of catalysts were pre-treated by Ar flow for 30 min. Initially, a flow of 50 *vol.%* CO_2/Ar was introduced and the DRIFTS signals were collected at 350 °C every 1 min. Subsequently, the 50 *vol.%* CO_2/Ar gas was switched to a flow of 50 *vol.%* CH_4/Ar. The DRIFTS signals were further collected at 350 °C every 1 min.

CO_2-CH_4 pulse chemistry experiments

CO_2-CH_4 pulse chemisorption experiments were performed on a Micromeritics AutoChem II 2920 instrument. Prior to the experiment, the catalyst (100 mg) was treated with Ar for 1 h at 200 ℃ and then heated to 380 °C. 10 *vol.%* CO_2/He was repeatedly introduced into the reactor until saturation of CO_2 adsorption. Subsequently, 10 *vol.%* CH_4/He was introduced into the reactor until saturation of CH_4 adsorption. The volume of the quantitative ring was 0.50 mL each time.

***In situ* mass spectrometry analysis**

First, the $Pt_{cluster}/CeO_2$-FLP catalysts were treated with $H_2^{18}O$ at 150 °C for 30 mins. Then, the $H_2^{18}O$ flow was terminated and replaced by Ar flow to remove physically adsorbed $H_2^{18}O$ molecules. After a 30 mins purge, the reactor temperature was increased from 200 °C at a 10 *vol.%* CH_4/He flow rate (50 mL min^{-1}). The desorption products were also analyzed online by MS. The m/z of the desorption products were: H_2 (*m/z*=2), CO (*m/z*=28) and $C^{18}O$ (*m/z*=30).

Reaction order calculations

The reaction orders of different reactants are determined by the reaction rate equation of DRM, as follows:

$$r_{CO} = k \cdot P^{\alpha}{}_{CH_4} \cdot P^{\beta}{}_{CO_2}$$

In the formula, r_{CO} represents the rate of the CO generation, k is the reaction rate constant, P_{CO2} represents the partial pressure of CO_2 under the reaction conditions, P_{CH4} represents the partial pressure of CH_4 under the reaction conditions, α represents the reaction order of CH_4, β represents the reaction order of CO_2.

DFT calculation

All periodic DFT calculations were performed using the Vienna Ab Initio Simulation Package (VASP).[1-3] The exchange-correlation energies were calculated using a generalized gradient approximation (GGA) with a Perdew-Burke-Ernzerhof (PBE) functional. The valence electron density is described by a plane wave basis set with an energy cut-off of 400 eV.[4] To treat the on-site Coulomb and exchange interaction of the strongly localized Ce 4f electrons, we used the DFT + U method with an effective U = 5 eV. We also considered the van der Waals dispersion forces between adsorbates and surfaces using the zero damping DFT-D3 method of Grimme.

The $CeO_2(110)$ surface contained five layers, the bottom three layers were fixed at the lattice position, and the top two layers were fully relaxed. The surface slab was separated by a vacuum height of 15 Å in the z-direction to eliminate unphysical interactions between periodic surface plates. Brillouin-zone integrations were performed using a Γ-centered (1 × 2 × 1) *k*-point mesh. The relaxation of the atomic structure is performed by using the conjugate gradient algorithm implemented in the VASP code or the quasi-Newtonian scheme until the forces and energies on all unconstrained atoms are less than 0.02 eV/Å and 10^{-4} eV.

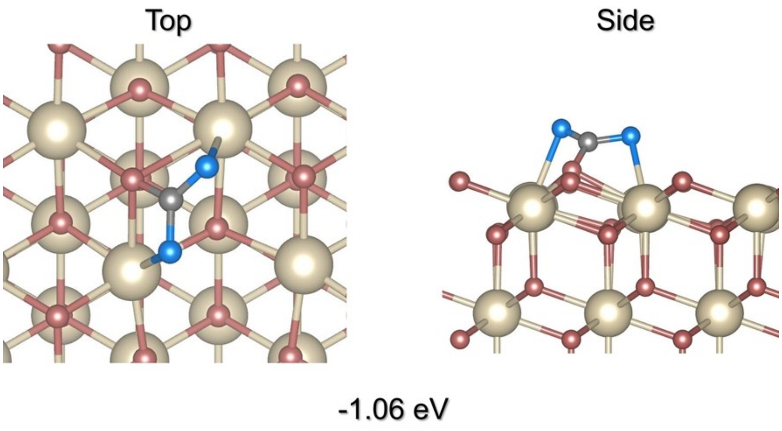

Figure S1. The adsorption behavior of CO_2 on CeO_2(111) with one oxygen vacancy.

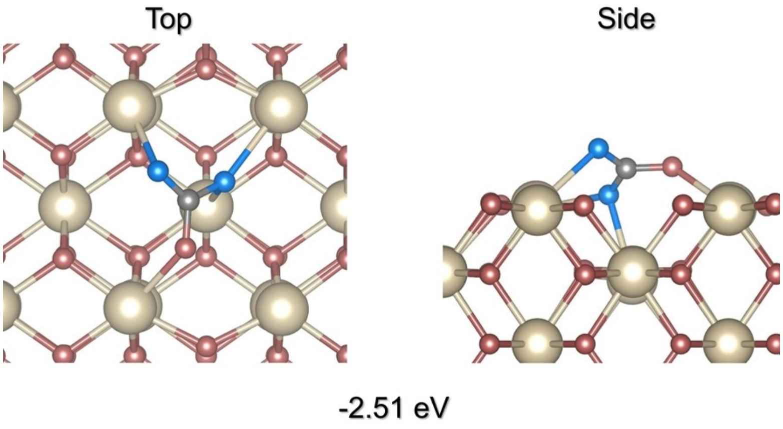

Figure S2. The adsorption behavior of CO_2 on CeO_2(110) with one oxygen vacancy.

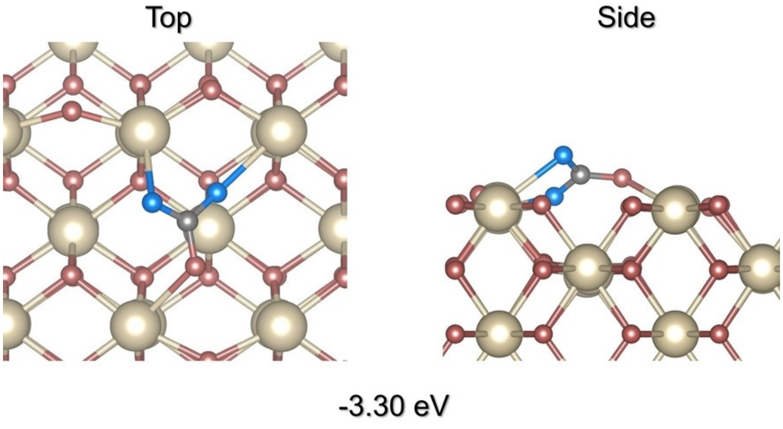

Figure S3. The adsorption behavior of CO_2 on the FLPs sites of CeO_2(110)-2O_V.

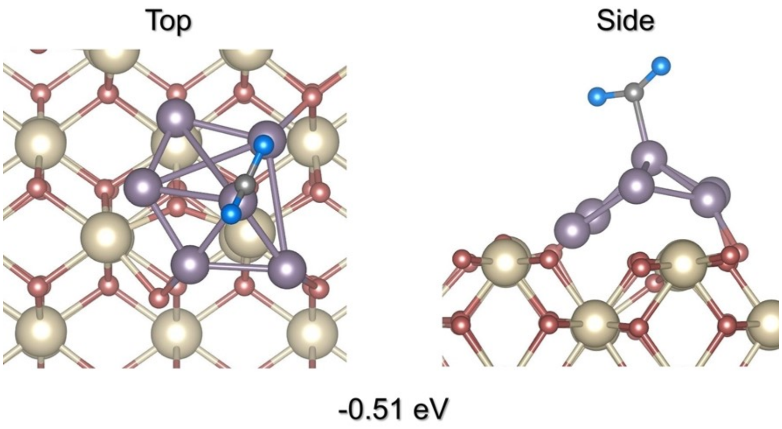

Figure S4. The adsorption behavior of CO_2 on the Pt_6 sites.

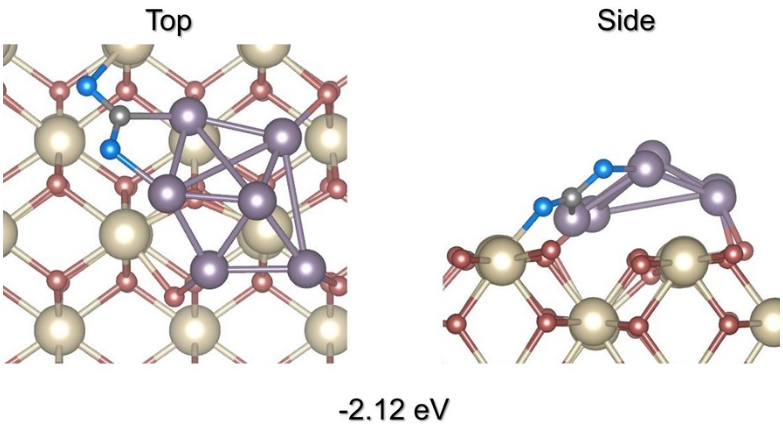

Figure S5. The adsorption behavior of CO_2 on the Pt_6-CeO_2 interface sites.

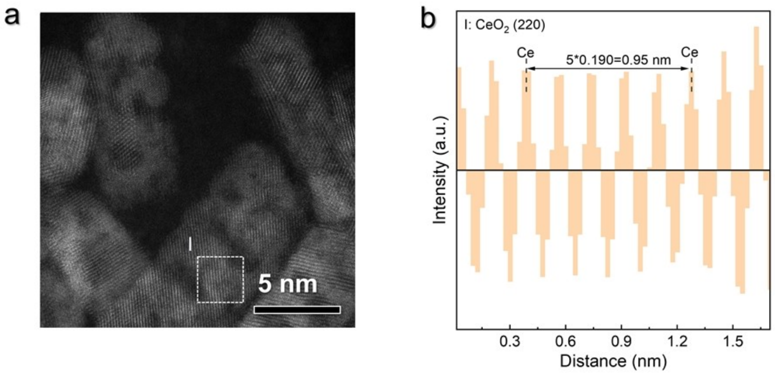

Figure S6. Characterization of the CeO_2-FLP supports. (a) HAADF-STEM image of CeO_2-FLP catalyst and (b) results of fast Fourier transform (FFT) post-crystallographic spacing of CeO_2-FLP catalyst.

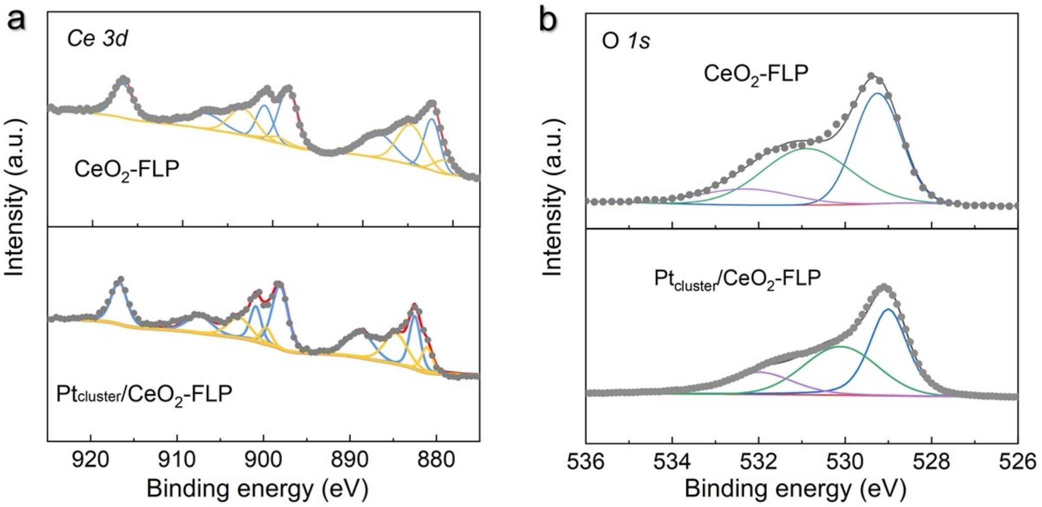

Figure S7. XPS analysis. XPS analysis of (**a**) Ce *3d* and (**b**) O *1s* peaks of CeO_2-FLP and $Pt_{cluster}/CeO_2$-FLP.

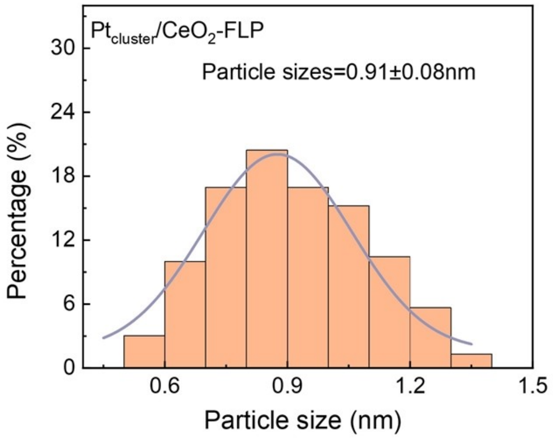

Figure S8. Size distribution of Pt clusters on the $Pt_{cluster}/CeO_2$-FLP catalysts.

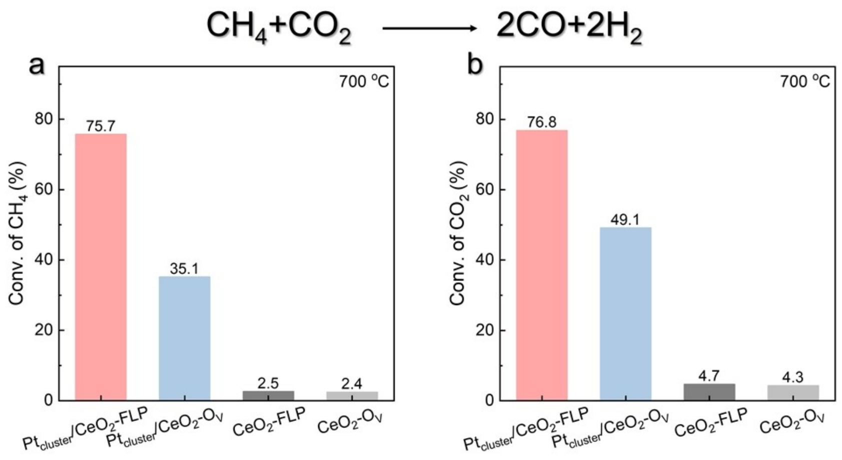

Figure S9. Catalytic performance of various catalysts with a WHSV of 30,000 mL g_{cat}^{-1} h^{-1} (CH_4:CO_2:N_2=2:2:1) at 700 °C. (a) CH_4 conversion and (b) CO_2 conversion.

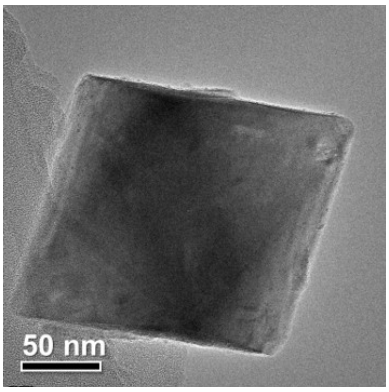

Figure S10. TEM image of the CeO_2-O_V supports.

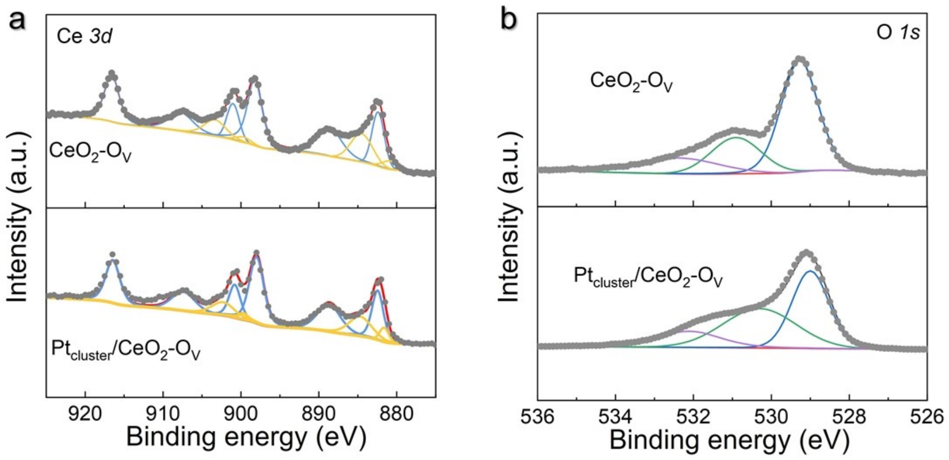

Figure S11. XPS analysis. (**a**) Ce *3d* and (**b**) O *1s* peaks of CeO_2-O_V and $Pt_{cluster}$/CeO_2-O_V.

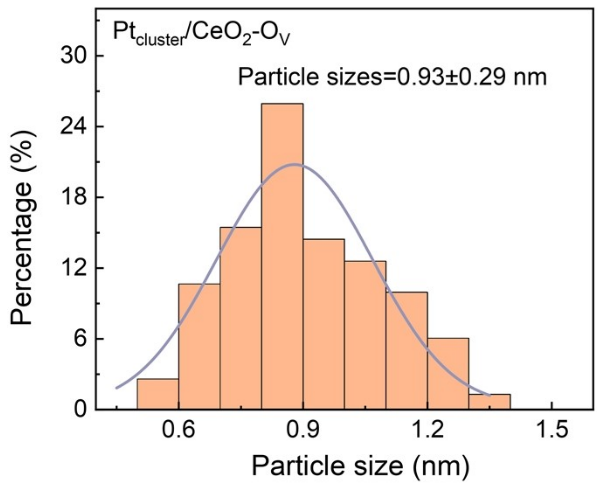

Figure S12. Size distribution of Pt clusters for the Pt$_{cluster}$/CeO$_2$-O$_V$ catalysts.

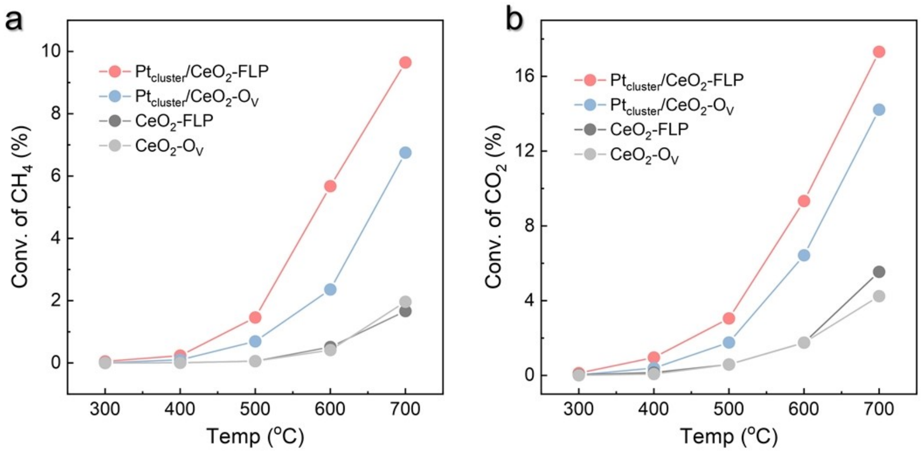

Figure S13. Catalytic performance of the DRM reaction. (**a**) CH_4 and (**b**) CO_2 conversions as function of reaction temperatures. **Reaction conditions:** 20 mg of catalysts, 60 mL min^{-1} gas flow (CH_4: 30 mL min^{-1}, CO_2: 30 mL min^{-1}), WHSV=180,000 mL g^{-1} h^{-1}.

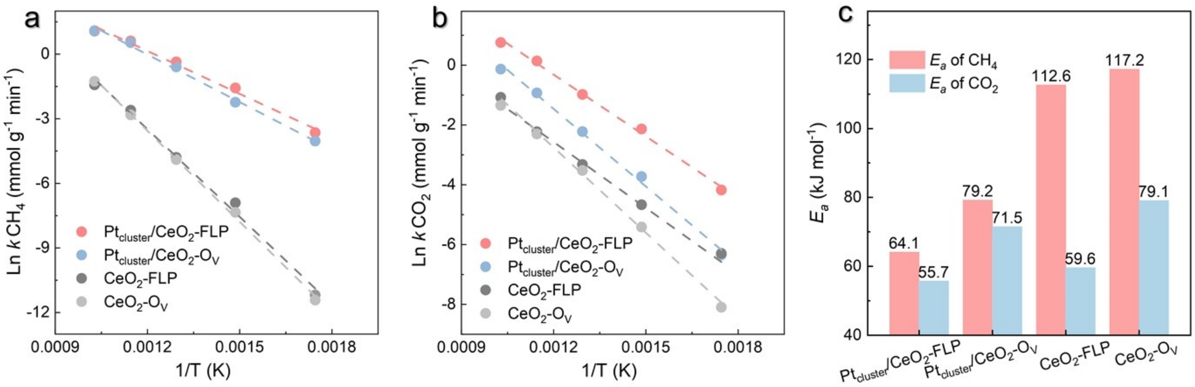

Figure S14. Correlation between Ln *k* and 1/*T*. Ln *k* derived from (**a**) CH_4 and (**b**) CO_2 conversion rates as a function of 1/*T*. (**c**) Calculated E_a values of various catalysts for DRM.

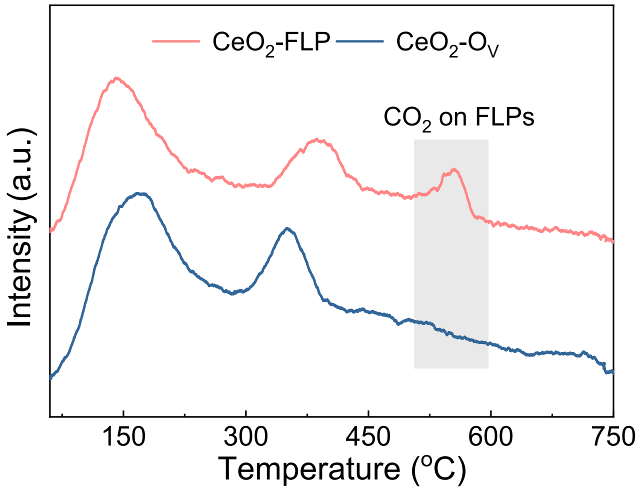

Figure S15. CO_2-TPD curves of the CeO_2-FLP and CeO_2-O_V catalysts.

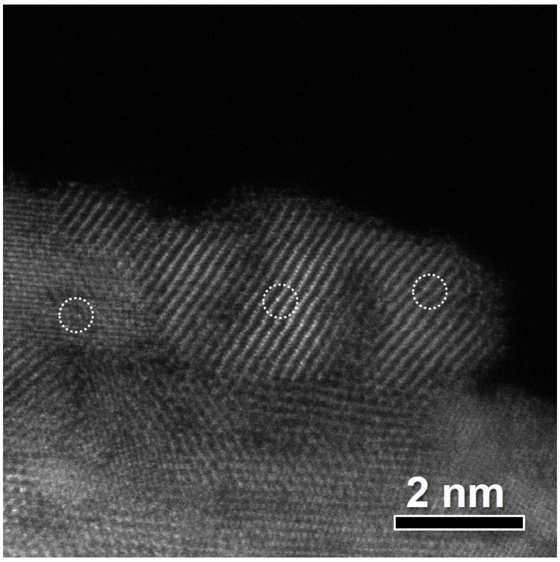

Figure S16. HAADF-STEM images of the Pt_1/CeO_2-FLP catalysts.

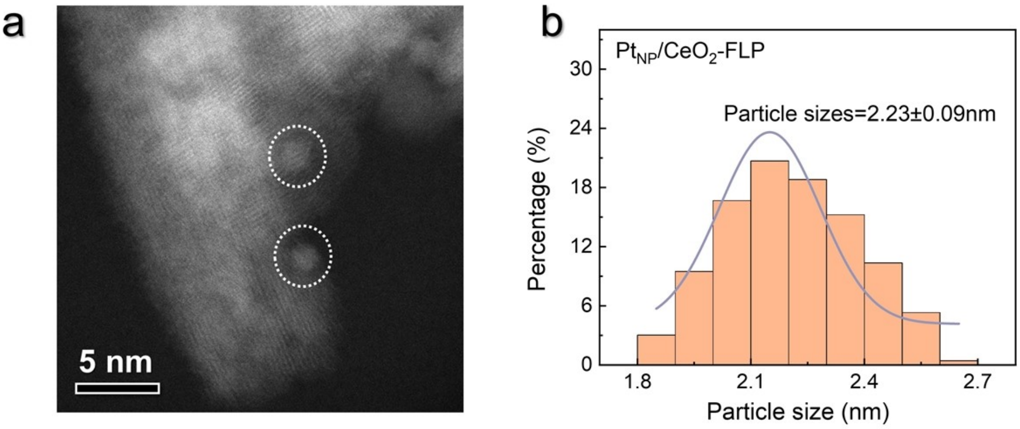

Figure S17. Characterization of Pt_{NP}/CeO_2-FLP. (**a**) HAADF-STEM image and (**b**) size distribution of Pt particle for the Pt_{NP}/CeO_2-FLP catalysts.

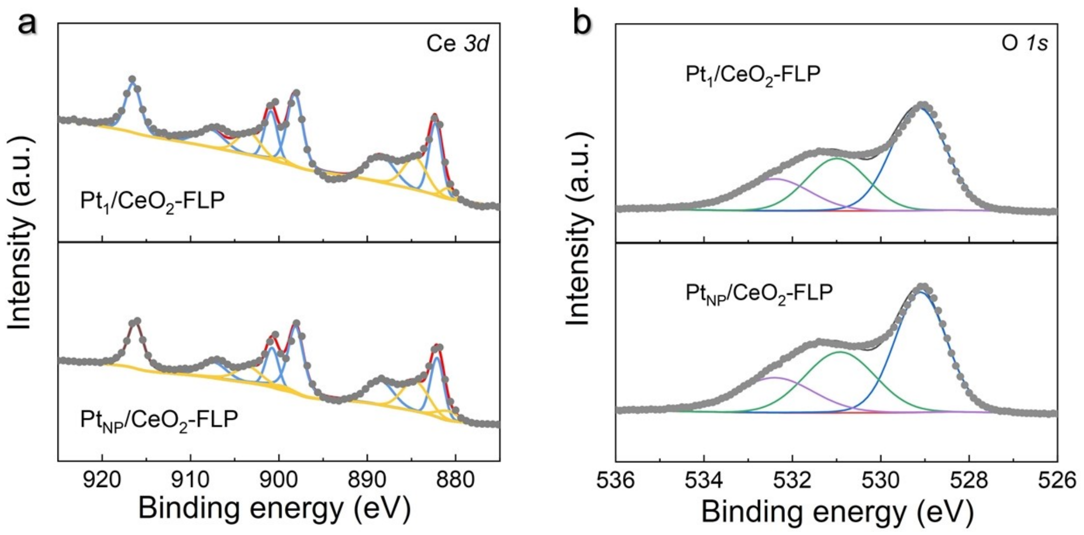

Figure S18. XPS analysis. (**a**) Ce 3*d* and (**b**) O 1*s* peaks of the Pt_1/CeO_2-FLP and Pt_{NP}/CeO_2-FLP catalysts.

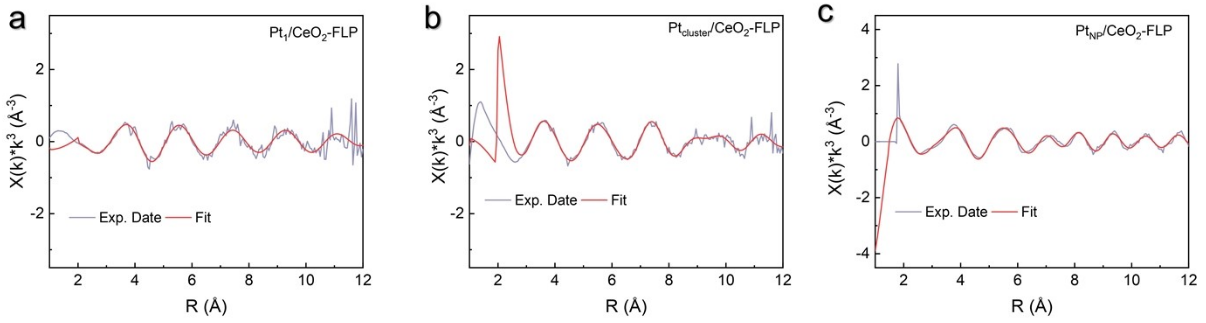

Figure S19. *K*-space of Pt *L*-edge EXAFS fitting curves of various catalysts without phase correction. (**a**) Pt_1/CeO_2-FLP, (**b**) $Pt_{cluster}/CeO_2$-FLP and (**c**) Pt_{NP}/CeO_2-FLP.

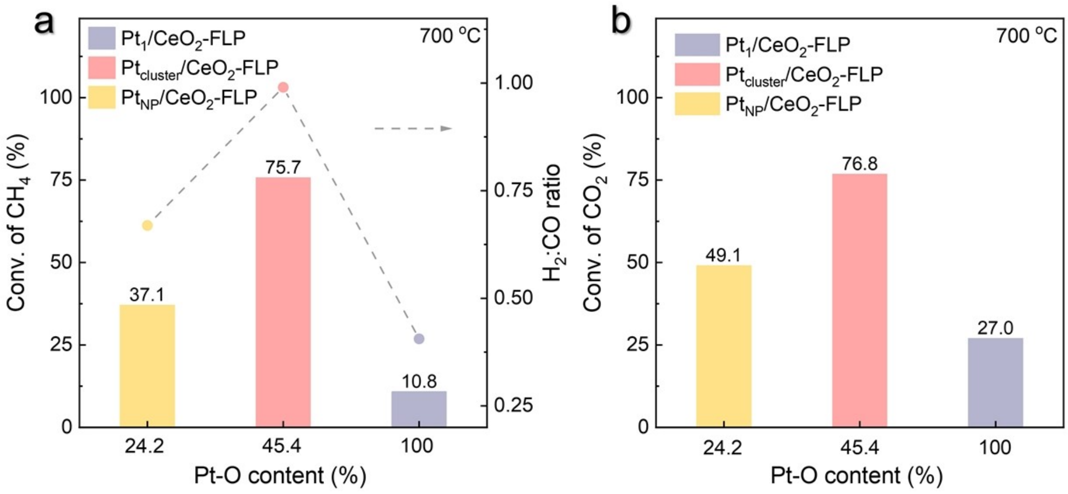

Figure S20. Catalytic performance of the Pt$_1$/CeO$_2$-FLP, Pt$_{cluster}$/CeO$_2$-FLP and Pt$_{NP}$/CeO$_2$-FLP catalysts. (**a**) CH_4 conversion and H_2:CO ratios. (**b**) CO_2 conversion.

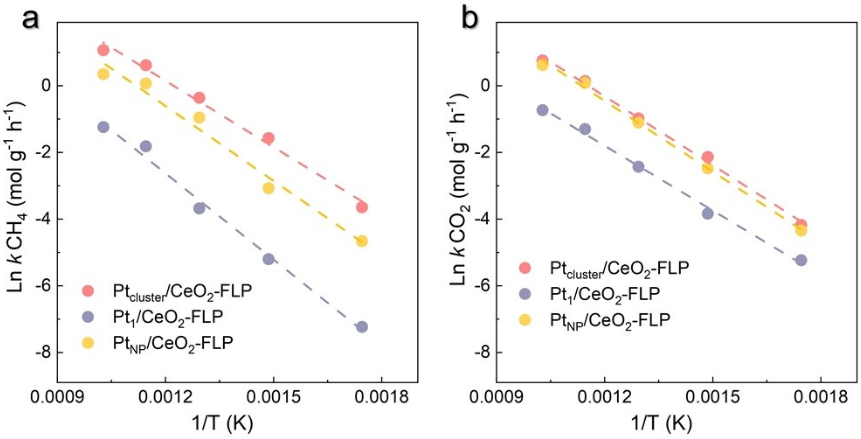

Figure S21. Correlation between Ln *k* and 1/*T*. Ln *k* derived from (**a**) CH_4 and (**b**) CO_2 conversion rates as a function of 1/*T* by the Pt$_1$/CeO$_2$-FLP, Pt$_{cluster}$/CeO$_2$-FLP and Pt$_{NP}$/CeO$_2$-FLP catalysts.

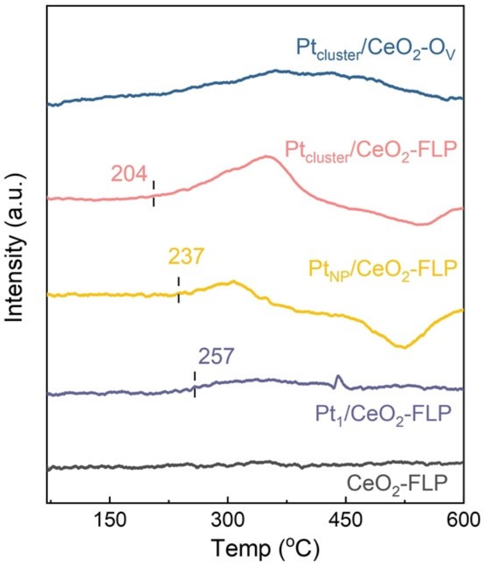

Figure S22. CH_4-TPR curves. CH_4-TPR profiles of the Pt$_{cluster}$/CeO$_2$-FLP, Pt$_1$/CeO$_2$-FLP, Pt$_{cluster}$/CeO$_2$-O$_V$, Pt$_{NP}$/CeO$_2$-FLP and CeO$_2$-FLP catalysts.

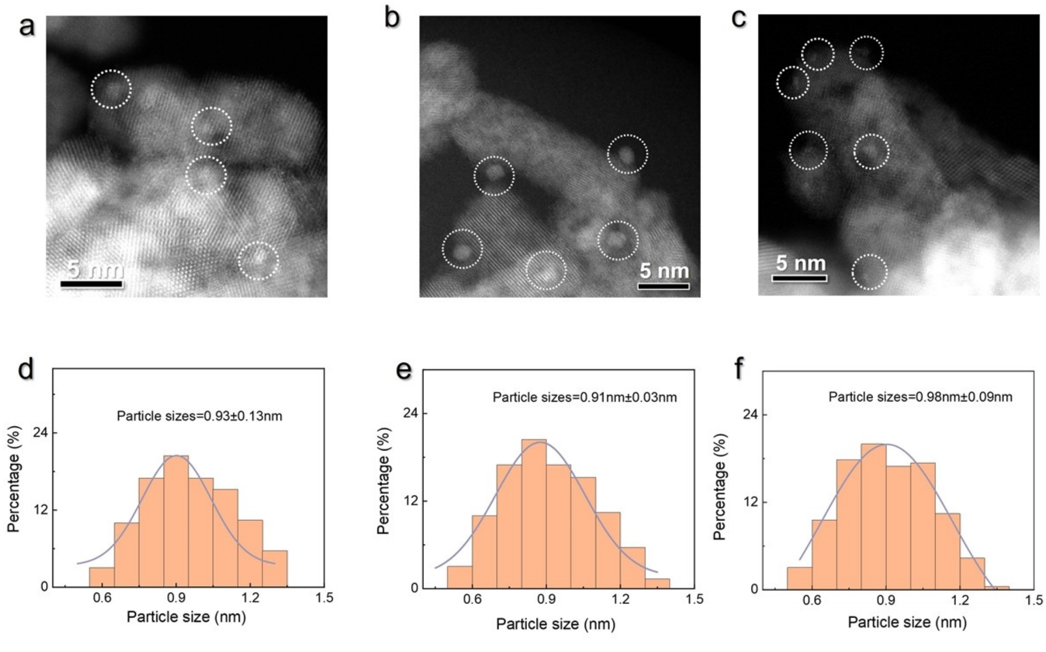

Figure S23. Characterization of Pt$_{cluster}$/CeO$_2$-FLP. HAADF-STEM images of Pt$_{cluster}$/CeO$_2$-FLP with Pt loadings of (**a**) 0.5 *wt*%, (**b**) 1.0 *wt*% and (**c**) 2.0 *wt*%. The Pt size distributions of Pt$_{cluster}$/CeO$_2$-FLP with Pt loadings of (**d**) 0.5 *wt*%, (**e**) 1.0 *wt*% and (**f**) 2 *wt*%.

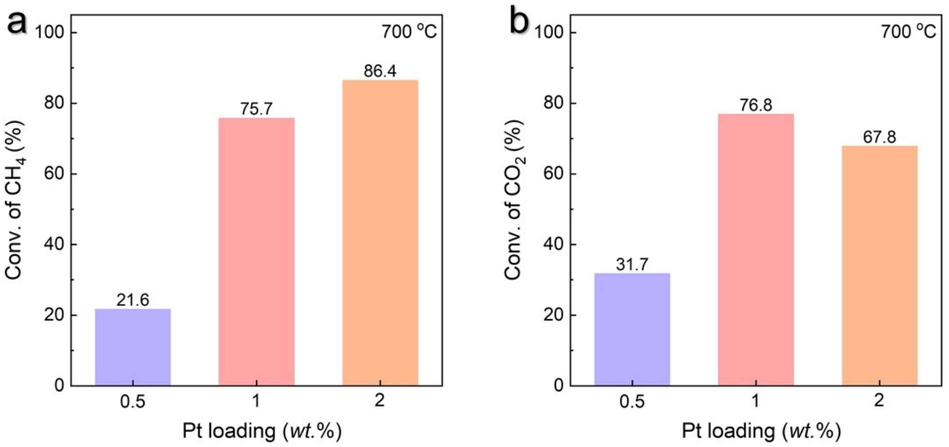

Figure S24. Catalytic performance of $Pt_{cluster}/CeO_2$-FLP with a WHSV of 30,000 mL g_{cat}^{-1} h^{-1} (CH_4:CO_2:N_2=2:2:1) at 700 °C. (**a**) CH_4 and (**b**) CO_2 conversions.

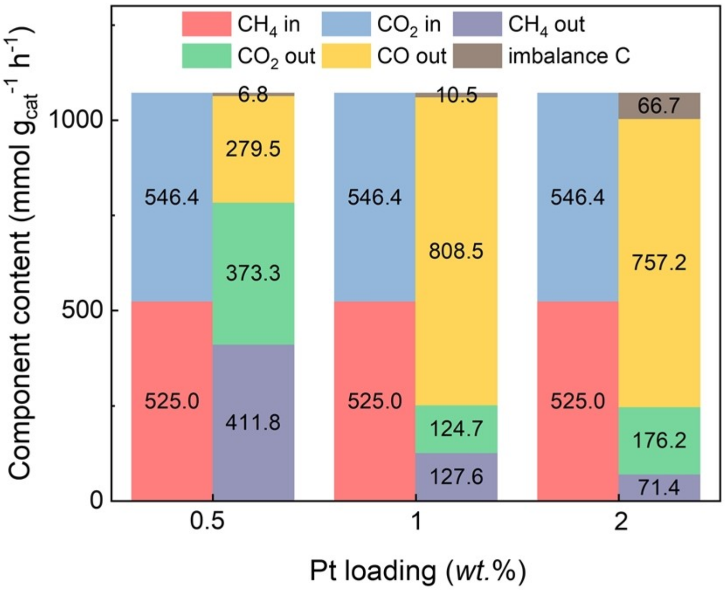

Figure S25. Carbon balance. Carbon balance data for various catalysts before and after the reaction with a WHSV of 30,000 mL g_{cat}^{-1} h^{-1} (CH_4:CO_2:N_2=2:2:1) at 700 °C.

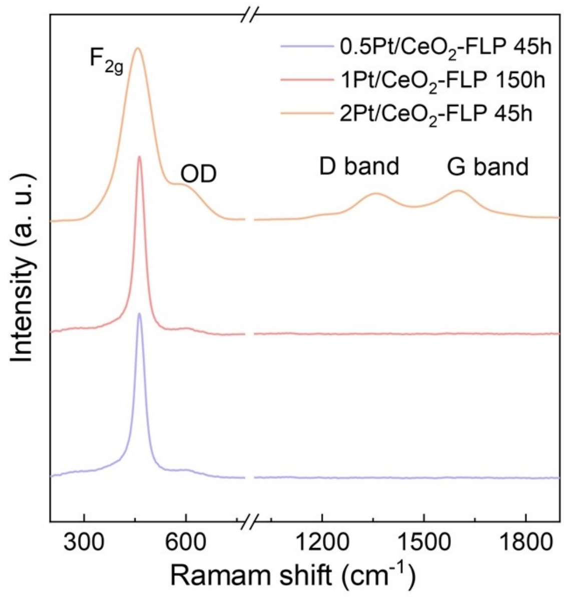

Figure S26. Raman spectra. Raman spectra of Pt/CeO_2-FLP with various Pt loadings after stability testing at 700 °C.

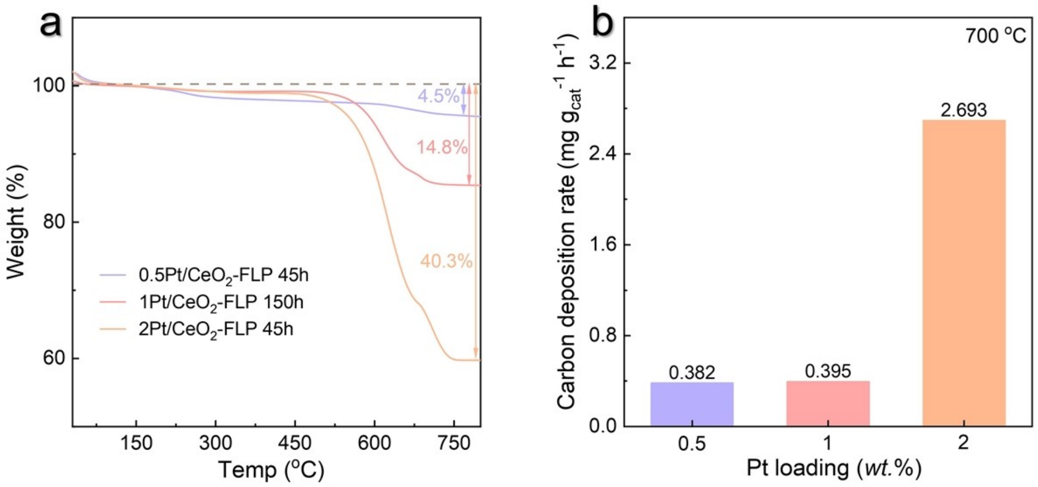

Figure S27. Carbon deposit analysis. (**a**) TGA profiles of Pt/CeO_2-FLP catalysts with various Pt loadings after test for various reaction times at 700 °C. (**b**) Carbon deposition rate of Pt/CeO_2-FLP catalysts with various Pt loadings after test at 700 °C.

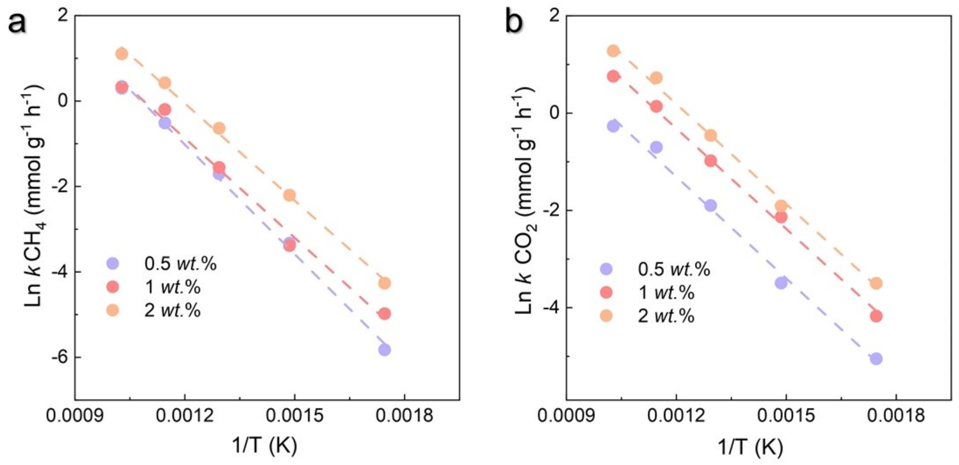

Figure S28. Correlation between Ln *k* and 1/*T*. Ln *k* derived from (**a**) CH_4 and (**b**) CO_2 conversion rate as a function of 1/*T* by various Pt/CeO_2-FLP catalysts.

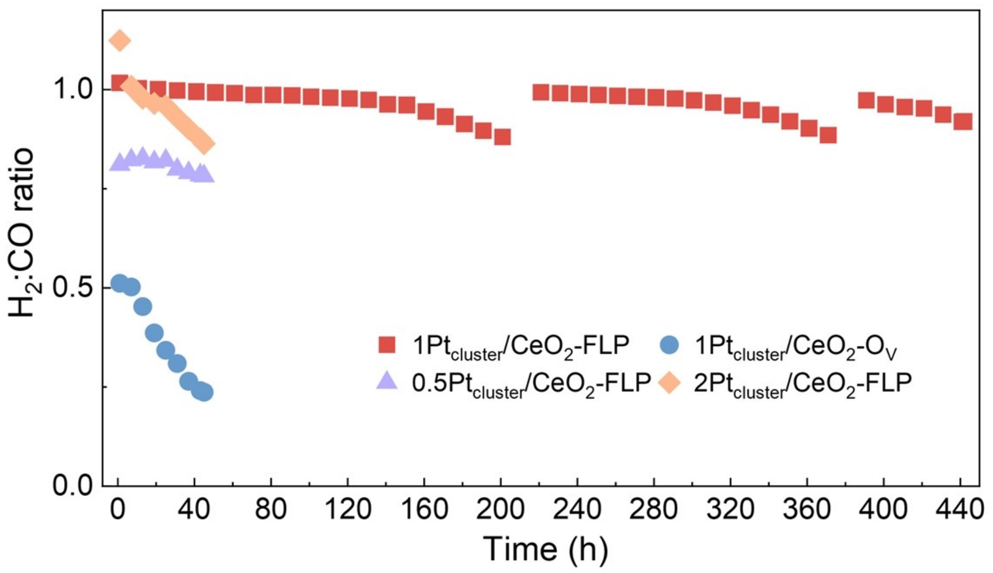

Figure S29. Catalytic stability. H_2:CO ratios of various catalysts with a WHSV of 30,000 mL g_{cat}^{-1} h^{-1} (CH_4:CO_2:N_2=2:2:1) at 700 °C.

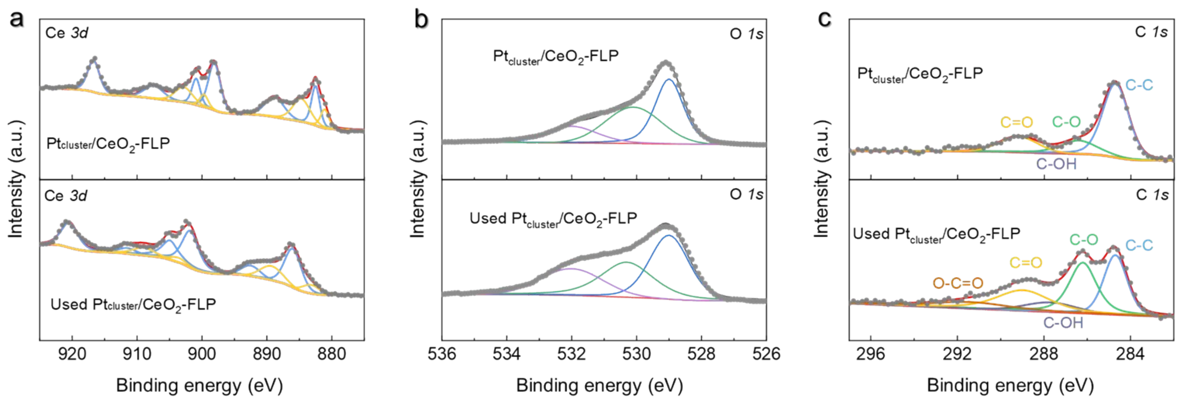

Figure S30. XPS analysis. (**a**) Ce 3*d*, (**b**) O 1*s* peaks and (**c**) C 1*s* peaks of the $Pt_{cluster}/CeO_2$-FLP and Used $Pt_{cluster}/CeO_2$-FLP catalysts.

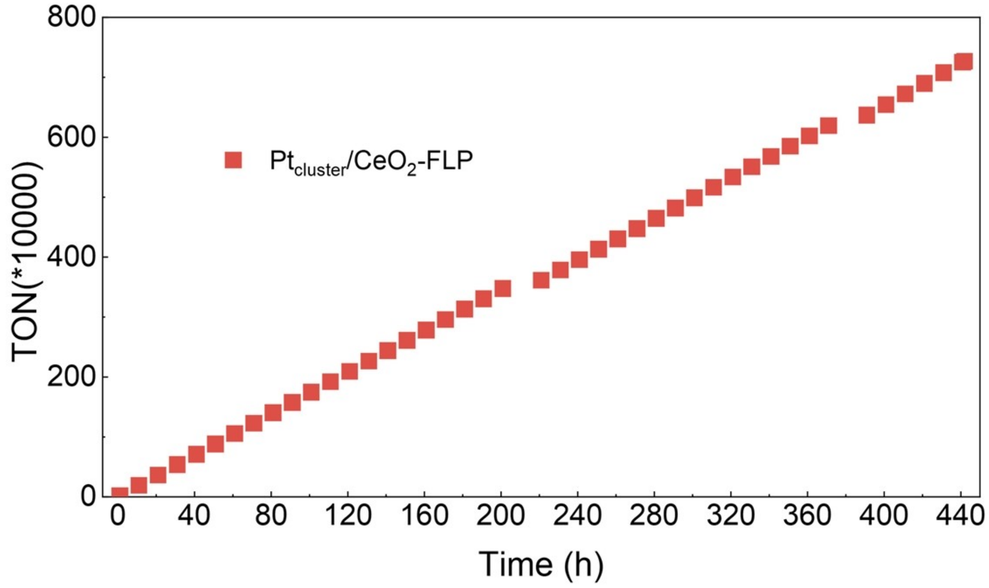

Figure S31. Catalytic stability. Turnover number of CH_4 conversions by the $Pt_{cluster}/CeO_2$-FLP catalysts for the DRM reaction at 700 °C.

Table S1. Summary of the structural properties of various catalysts.

Sample	Pt loading (*wt*%)	Pt Size (nm)	Dispersion (%)	Ce^{3+} fraction (%)	Ce^{3+}-O fraction (%)
CeO_2-FLP	-	-	-	29.4	52.4
CeO_2-O_V	-	-	-	19.3	40.3
$Pt_{cluster}$/CeO_2-FLP	0.91	0.9 ± 0.1	43.2	30.9	55.2
$Pt_{cluster}$/CeO_2-O_V	1.1	0.9 ± 0.3	48.2	20.4	42.4
Pt_{NP}/CeO_2-FLP	1.0	2.2 ± 0.1	15.2	30.1	54.2
Pt_1/CeO_2-FLP	0.45	-	99.5	29.8	53.7

Table S2. Summary of catalytic performance of the DRM reaction.

Catalysts	Temperature (^oC)	WHSV (mL g^{-1} h^{-1})	CH_4 Conv. (%)	CH_4 Conv. Rate (mol g_M^{-1} h^{-1})	TOF (h^{-1})	Stability (h)	H_2:CO ratio	Ref.
**$Pt_{cluster}/CeO_2$-FLP**	**700**	**30,000**	**75.7**	**93.90**	**18310.5**	**440**	**0.99**	**This work**
NiMo/MgO	650	60,000	52	2.53	146.74	850	0.82	[5]
NiMo/MgO	750	60,000	82	3.99	231.42	850	0.93	[5]
NiMo/MgO $_2$	850	60,000	100	4.87	282.46	850	0.99	[5]
$Ru_{1.5}/Ni_1$-MgO	650	60,000	62	11.07	1118.07	1200	0.71	[6]
$Ru_{1.5}/Ni_1$-MgO	700	60,000	77	13.75	1388.75	1200	0.81	[6]
$Ru_{1.5}/Ni_1$-MgO	750	60,000	83	14.82	1496.82	1200	0.93	[6]
$Ru_{1.5}/Ni_1$-MgO	800	60,000	86	15.35	1550.35	1200	0.98	[6]
Ni/CeO_2-SiO_2	800	54,000	82	17.79	1031.82	2000	0.92	[7]
Ru/LaO_x-SiO_2	700	100,000	72	64.28	6492.28	400	0.92	[8]
Ru/LaO_x-SiO_2	750	100,000	83	74.10	7484.1	400	0.96	[8]
Ru/LaO_x-SiO_2	800	100,000	92	82.14	8296.14	400	0.98	[8]
Pt-CeO_2	400	6,000	14	2.08	405.6	10	0.68	[9]
Pt-CeO_2	500	6,000	21	3.12	608.4	10	0.83	[9]
Pt-CeO_2	600	6,000	51	7.58	1478.1	10	0.86	[9]
$PtCo/CeO_2$	600	240,000	25.5	20.45	3987.75	13	0.71	[10]
Ni/CeO_2	700	36,000	/	1.51	87.58	8	0.86	[11]
$Ni/Ce_{0.9}Eu_{0.1}O_{1.95}$	500	60,000	8	7.78	451.24	12	0.76	[12]
$Ni/Ce_{0.9}Eu_{0.1}O_{1.95}$	600	60,000	23	22.38	1298.04	12	0.82	[12]
Pt/CeO_2	500	30,000	11.1	3.72	725.4	70	0.72	[13]
Pt/CeO_2	600	30,000	31.4	10.53	2053.35	70	0.79	[13]
Pt/CeO_2	700	30,000	53.6	17.98	3506.1	70	0.85	[13]
Pt/CeO_2	800	30,000	77.3	25.94	5058.3	70	0.90	[13]
NiYAl	550	15,000	-	0.19	11.02	-	0.78	[14]
Ni/ZrO_2	600	72,000	15	0.64	37.12	10	0.85	[15]
Ni/ZrO_2	700	72,000	26	1.37	79.46	10	0.87	[15]

Ni/ZrO_2	800	72,000	38	2.82	163.56	10	0.85	[15]
$NiAl_2O_4$	500	14,400	15	3.77	218.66	50	0.86	[16]
$NiAl_2O_4$	600	14,400	32	3.98	230.84	50	0.87	[16]
$NiAl_2O_4$	700	14,400	66	0.05	2.9	50	0.84	[16]
$NiAl_2O_4$	800	14,400	88	0.11	6.38	50	0.83	[16]
$NiAl_2O_4$	900	14,400	93	0.16	9.28	50	0.87	[16]
Ni-Mo/Al_2O_3	600	20,000	28	4.90	284.2	8	1.05	[17]
Ni-Mo/Al_2O_3	700	20,000	66	0.72	41.76	8	0.57	[17]
Ni-Mo/Al_2O_3	800	20,000	92	1.31	75.98	8	0.66	[17]
Ni/ZrO_2-Al_2O_3	800	42,000	93	2.61	151.38	7	0.72	[18]
20Mo2Ni	650	10,800	6	4.71	273.18	-	0.70	[19]
20Mo2Ni	700	10,800	11	1.57	91.06	-	0.70	[19]
20Mo2Ni	750	10,800	22	4.94	286.52	-	0.78	[19]
20Mo2Ni	800	10,800	39	45.8	2656.4	-	0.68	[19]
$Ni_2Al_2O_5$	700	52,400	40	2.8	162.4	-	0.86	[20]

Reference:

[1] Kresse. G., Furthmüller. J, *Comput. Mater. Sci.***1996,** 6, 15-50.

[2] Kresse. G., Hafner. J, *Phys. Rev. B* **1993,** 47, 558-561.

[3] Kresse. G., Furthmüller. J, *Phys. Rev. B* **1996,** 54, 11169-11186.

[4] Kresse. G., Joubert. D, *Phys. Rev. B* **1999,** 59, 1758-1775.

[5] Y. Song, E. Ozdemir, S. Ramesh, A. Adishev, S. Subramanian, A. Harale, M. Albuali, B.A. Fadhel, A. Jamal, D. Moon, S.H. Choi, C.T. Yavuz, *Science*, **2020,** 367, 777-781.

[6] J. Yang, Z. Cao, Y. Wan, S. Guan, B. Jiang, Y. Yamauchi, H. Li, *Adv. Energy. Mater.* **2024,** 202404936.

[7] Y. Li, Z. Li, N. Wang, Y. Zha, K. Zheng, Y. Xu, B. Liu, X. Liu, *Chem Catal.* **2024,** 5, 101189-101208.

[8] T. Zhou, X. Li, J. Zhao, L. Luo, Y. Wang, Z. Xiao, S. Hu, R. Wang, Z. Zhao, C. Liu, W. Wu, H. Li, Z. Zhang, L. Zhao, H. Yan, J. Zeng, *Nat. Mater.* **2025,** 24, 891-899.

[9] D. Shen, Z. Li, J. Shan, G. Yu, X. Wang, Y. Zhang, C. Liu, S. Lyu, J. Li, L. Li, *App. Catal. B: Environ.* **2022,** 318, 121809-121820.

[10] Z. Xie, B. Yan, S. Kattel, J.H. Lee, S. Yao, Q. Wu, N. Rui, E. Gomez, Z. Liu, W. Xu, L. Zhang, J.G. Chen, *App. Catal. B: Environ.* **2018,** 236, 280-293.

[11] Q. Zhang, M. Mao, Y. Li, Y. Yang, H. Huang, Z. Jiang, Q. Hu, S. Wu, X. Zhao, *App. Catal. B: Environ.* **2018,** 239, 555-564.

[12] Y. Wang, R. Zhang, B. Yan, *J. Catal.*, **2022,** 407, 77-89.

[13] Z. Zhang, J. Li, W. Gao, Y. Ma, Y. Qu, *J. Mater. Chem. A*, **2015,** 3, 18074-18082.

[14] S. Imada, X. Peng, Z. Cai, A. Najib, M. Miyauchi, H. Abe, T. Fujita, *Materials*, **2020,** 13, 2044-2051.

[15] S. Azeem, R. Aslam, M. Saleem, H. Soon Min, *Int. J. Chem. Eng.*, **2022,** 2022, 1-13.

[16] S. Ali, M.M. Khader, M.J. Almarri, A.G. Abdelmoneim, Ni-based nano-catalysts for the dry reforming of methane, *Catal. Today*, **2020,** 343, 26-37.

[17] L. Yao, M.E. Galvez, C. Hu, P. Da Costa, *Int. J. Hydrog. Energy* **2017,** 42, 23500-23507.

[18] A.S. Al-Fatesh, A.A. Ibrahim, A.I. Osman, A.E. Abasaeed, M.F. Alotibi, S.A. Alfatesh, D.W. Rooney, A.H. Fakeeha, C.Y. Yin, , *Energy Sci. & Eng.* **2023,** 11, 3780-3789.

[19] L. Dehimi, M. Gaillard, M. Virginie, A. Erto, Y. Benguerba, *Int. J. Hydrog. Energy* **2016,** 45, 24657-24669.

[20] J.L. Rogers, M.C. Mangarella, A.D. D'Amico, J.R. Gallagher, M.R. Dutzer, E. Stavitski, J.T. Miller, C. Sievers, *ACS Catal.*, **2016,** 6, 5873-5886.
